# The InterModel Vigorish (IMV) as a flexible and portable approach for quantifying predictive accuracy with binary outcomes

**DOI:** 10.1371/journal.pone.0316491

**Published:** 2025-03-21

**Authors:** Benjamin W. Domingue, Charles Rahal, Jessica Faul, Jeremy Freese, Klint Kanopka, Alexandros Rigos, Ben Stenhaug, Ajay Shanker Tripathi

**Affiliations:** 1 Graduate School of Education, Stanford University, Stanford, California, United States of America; 2 Demographic Science Unit and Nuffield College, University of Oxford, Oxford, United Kingdom; 3 Michigan Center on the Demography of Aging, University of Michigan, Ann Arbor, Michigan, United States of America; 4 Department of Sociology, Stanford University, Stanford, California, United States of America; 5 Steinhardt School of Culture, Education, and Human Development, New York University, New York, New York, United States of America; 6 Institute for Futures Studies, Stockholm, Sweden,; 7 Department of Economics, Lund University, Lund, Sweden; 8 Department of Electrical Engineering, Stanford University, Stanford, California, United States of America; Sant’Anna School of Advanced Studies Institute of Management: Scuola Superiore Sant’Anna Istituto di Management, ITALY

## Abstract

Understanding the “fit” of models designed to predict binary outcomes has been a long-standing problem across the social sciences. We propose a flexible, portable, and intuitive metric for quantifying the change in accuracy between two predictive systems in the case of a binary outcome: the InterModel Vigorish (IMV). The IMV is based on an analogy to weighted coins, well-characterized physical systems with tractable probabilities. The IMV is always a statement about the change in fit relative to some baseline model—which can be as simple as the prevalence—whereas other metrics are stand-alone measures that need to be further manipulated to yield indices related to differences in fit across models. Moreover, the IMV is consistently interpretable independent of baseline prevalence. We contrast this metric with alternatives in numerous simulations. The IMV is more sensitive to estimation error than many alternatives and also shows distinctive sensitivity to prevalence. We consider its performance using examples spanning the social and natural sciences. The IMV allows for precise answers to questions about changes in model fit in a variety of settings in a manner that will be useful for furthering research and the understanding of social outcomes.

## 1 Introduction

There has been a recent increase in the use of methods focused on prediction and in the ‘fit’ of models [1], coinciding with calls for a closer integration of explanation and prediction more broadly [2]. An independent long-standing question involves understanding, evaluating and—perhaps most importantly—comparing the quality of predictions from models trained on binary outcomes. An array of (frequently related) techniques have been developed: the ROC curve [3], the harmonic mean of precision and recall (the *F*_1_ score [4]), other quantities related to the confusion matrix determined by a given decision rule [5], cross-entropy [6], and Information Criteria such as the Akaike and Bayesian Information Criteria [7]. This is in addition to various pseudo-*R*^2^ estimates [8].

The provisioning of accessible, intuitive, and portable metrics is essential to realizing the potential of machine intelligence and other predictive approaches across social science domains [9,10]. To that end, existing approaches have critical shortcomings. First, some metrics do not generalize given that they depend on sample-specific quantities, thus necessitating attempts to both generate sample size-sensitive benchmarks [11] and other attempts to reduce sample size dependency in related contexts [12]. Second, there is a lack of guidance about how to compare predictive gains relative to the base rate (i.e., the problem of “prevalence” or “imbalance”) of the outcome [13]. Third, most metrics are absolute statements about the fit of a given model. If interest is in a comparison between models, further manipulation of the metrics is frequently needed (and such manipulations may not be readily interpretable). Collectively, these limitations challenge our ability to make generalizable inferences about the quality of models used across various contexts both within and beyond the social sciences.

Statements about a single model have utility in many settings. For example, the evaluation of whether a black box diagnostic test is of sufficient accuracy to be used in a specific setting. In that case, something like the AUC can be interpreted alongside established benchmarks [14] (e.g., the clinical accuracy of COVID-19 tests [15]). However, such stand-alone approaches have limitations. Suppose there is an outcome *y* and two predictive systems *f * and *g*. A stand-alone metric (e.g., *R*^2^ as defined in the Fragile Families Challenge [16]: $R^{2}=1\;-\;\frac{\sum {\left(y_{i}-p_{i}\right)}^{2}}{\sum {\left(y_{i}-ȳ\right)}^{2}}$) produces an index of fit based on each system’s predictive accuracy, which we can denote as *m_f_* and *m_g_*. Predictions from *f * are better than those from *g* if *m_f_*>*m_g_*; indeed, much existing work stops there and just notes the direction of this inequality. But “how much better?” and “how does this relate to other applications?” are important and challenging questions that require answers if we are to maximize the impact of predictive social science and move towards a more coherent realization of external validity. Having a single numeric summary of the “difference” between *m_f_* and *m_g_* would allow us to better answer those questions, a topic of current interest within the social sciences [16,17]. Many existing metrics either do not allow for a summative comparison of *m_f_* and *m_g_* (e.g., AUC) or produce summaries that can be challenging to interpret given that the values depend on sample size (AIC) or may have an unclear dependence on $\hat{y}$ (*R*^2^).

This paper introduces a novel metric designed to overcome these challenges for use in predictive systems that generate predictions in the form of probabilities (c.f., class labels). It is based on translating the level of uncertainty for a given predictive system into a canonical physical system—a weighted coin—and then building inference around the well-characterized statistical properties of that physical system. This metric, the InterModel Vigorish (hereafter, IMV), generalizes across multiple predictive systems that may vary in outcome, predictors, and approaches to prediction (so long as the approach generates probabilities rather than classes). Note that this metric is discussed in the specific context of psychometric models for dichotomous item responses elsewhere [61]. In tying notions of profits from gambles to questions of prediction, this work ties into the deep traditions of early statisticians such as Pascal and Huygens [18] who used games of chance as a means to better understand probability.

## 2 The InterModel Vigorish

### 2.1 Introducing the IMV

**Fig 1:**
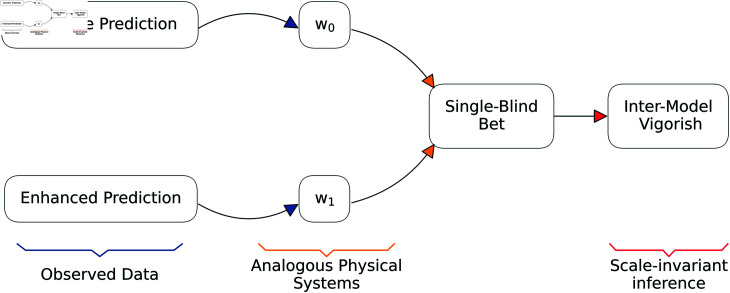
The IMV approach to quantifying prediction. Predictions are translated to an analogous physical system (weighted coins). The single-blind bet is then constructed based on payoff odds generated via *w*_0_ yet one player knows the true probability of success is *w*_1_. The IMV is constructed from the expected winnings associated with the side information contained in *w*_1_. Results can be compared across outcomes given that the fair bet is based on *w*_0_.

We focus on the problem of constructing a generalizable—in the sense that values of the IMV are comparable across outcomes—metric for comparing the accuracy of two predictive systems for binary outcomes. These are the ‘baseline’ and ‘enhanced’ predictions. These names are chosen to increase intuition as one might anticipate the enhanced prediction containing valuable ‘side’ information not available to the baseline prediction (but the enhanced prediction need not, in fact, be an improvement to the baseline prediction). At first glance, requiring two systems may seem restrictive. However, given that one of the models can be a prediction based on prevalence alone (i.e., the outcome’s mean), it is not (alternative approaches such as pseudo-*R*^2^ may use prevalence in a similar capacity). This approach is a multi-step process (see the schematic in Fig 1) described in detail below.

### 2.2 Defining the IMV

#### 2.2.1 Quantifying randomness

Suppose for *i* ∈ {1, ..., *n* } we have a vector $y\equiv \left(y_{i}\right)$ of observations of some binary outcome variable *Y * (so, $y_{i}\in \{0,1\}$ for each observation *i*). A *predictive system* *p* consists of a probabilistic prediction $p_{i}\in (0,1)$ for each observation *i* = 1 , … , *n*. The interpretation is that the system predicts that, for the *i*-th observation, *y_i_*=1 with probability *p_i_* and *y_i_*=0 with probability 1−*p_i_*. The likelihood assigned by the system to an observation *i* is $L_{i}\equiv p_{i}^{y_{i}}{\left(1-p_{i}\right)}^{1-y_{i}}$, while the log-likelihood of that observation is ℓi≡ log ⁡ (Li)=yi log ⁡ pi+(1−yi)log ⁡ (1−pi). To evaluate the system’s ability to predict the outcome variable, consider the system’s likelihood evaluated on the data set *y*:


$$\begin{array}{c} L(p;y)\equiv \overset{N}{\underset{i=1}{\prod }}p_{i}^{y_{i}}{\left(1-p_{i}\right)}^{1-y_{i}}. \end{array}$$ (1)

To make comparisons between models of different sample size, we take the geometric mean of an observation’s likelihood according to system *p*:


$$\begin{array}{c} A(p;y)\equiv L{\left(p;y\right)}^{1∕n}. \end{array}$$ (2)

Our goal is to identify a probability (or weight) of a weighted coin such that the expected log likelihood of the toss of a coin with that weight is the same as the mean log likelihood of the system *p* evaluated on data *y*. This weight *w* ( *p* ; *y* ) is given by:


w(p;y)≡ {w∈[1∕2,1]:wlog ⁡ w+(1−w)log ⁡ (1−w)=1n∑i=1nℓi}. (3)

**Fig 2:**
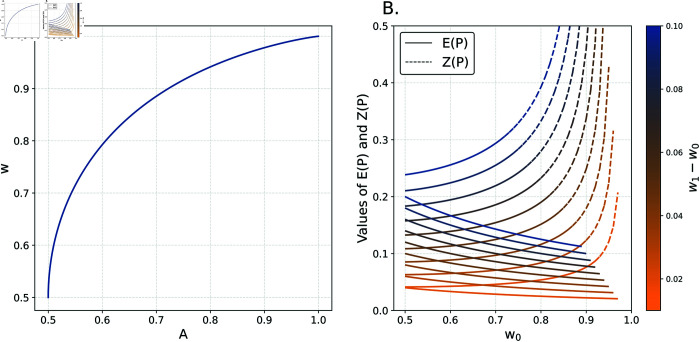
Properties of the IMV. Panel A shows a mapping between *A*and*w*. Panel B shows $E(P)\;\text{as}\;\text{a}\;\text{function}\;\text{of}\;w_{0}\;\text{and}\;w_{1}\;-\;w_{0}$.

For our purposes, a coin with weight 1 − *w* ( *p* ; *y* ) is equivalent to a coin with weight *w* ( *p* ; *y* ) ; we choose *w* ≥ 1 ∕ 2. The weight *w* ( *p* ; *y* ) describes the quality of predictions *p* in describing the observed *y*. Note also that the coin with weight *w* will be equivalent to the predictive system in terms of entropy [19]. A visualization of the curve linking *A* ( *p* ; *y* ) to *w* ( *p* ; *y* ) is shown in Fig 2 Panel A.

#### 2.2.2 A betting analogy

Consider a bet involving two parties, the house and a gambler. The parties wager over the outcome of a binary variable, which can be either positive or negative. The house bets $1 on the positive outcome and the gambler bets $1/O on the negative outcome. The party whose chosen outcome is realized wins the bet and takes the combined pot of $(1+1/O). This bet is *fair* if the house has zero expected gains should the gambler take up the wager. So, if the probability of a positive outcome is *w*, the bet is fair if


$$\begin{array}{c} w\times \frac{1}{O}-(1-w)\times 1=0. \end{array}$$ (4)

Thus, the bet will be fair if *O* is equal to the odds ratio, $O(w)\equiv \frac{w}{1-w}$.

Our method aims to compare the predictive accuracy between two different prediction models, the baseline and enhanced predictions. Consider the following scenario. Suppose that the house offers odds of $O(w_{0})$ where *w*_0_ is based on the baseline prediction. However, unbeknownst to the gambler, the probability of the positive outcome is actually *w*_1_ where *w*_1_ is the weight of the coin associated with the enhanced prediction. In this scenario, the bet is not fair if $w_{0}\neq w_{1}$. Should the gambler take up the bet, the house expects to win


$$\begin{array}{c} \;w_{1}\times \frac{1}{O(w_{0})}-(1-w_{1})\times 1=\frac{w_{1}-w_{0}}{w_{0}}. \end{array}$$ (5)

This amount is known as the house’s edge or the ‘vigorish’ of the bet. The IMV (or $\frac{w_{1}-w_{0}}{w_{0}})$ takes values in [-1,1] as $w_{0},w_{1}\in [0.5,1]$.

To define the IMV for predictions *p*^0^ and *p*^1^ for data *y*, we first identify $w_{0}=w(p^{0};y)$ and $w_{1}=w(p^{1};y)$ via Eq 3. The IMV—denoted as $\omega (p^{0},p^{1};y)$—is defined as


$$\begin{array}{c} \omega (p^{0},p^{1};y)\equiv \frac{w_{1}-w_{0}}{w_{0}}. \end{array}$$ (6)

It is the house’s vigorish supposing that the probability of the positive outcome was $w(p^{1},y)$ yet the house was offering odds based on $w(p^{0},y)$.

### 2.3 Computing the IMV

#### 2.3.1 A toy example in R

We can compute the IMV in a simple example so as to develop intuition for this quantity. Code to reproduce this example in R is shown below with equivalent examples in Python and MATLAB available in the Supporting Information (S1-II.1). Suppose we produce outcomes via a combination of 20 tosses of a fair coin (probability of 0.5 for heads) and 20 tosses of a heavily weighted coin (probability of 0.95 for heads). Researchers would be blind to this information about the weights of the coin in general. The fair coin produces 14 heads and the weighted coin produces 19 heads; thus our observed data is 33 heads and 7 tails. We do not attempt to quantify the level of randomness in this data; randomness, for our purposes, is only defined in the context of a specific model for the data generating process. As an illustration, suppose one has a set of heads and tails. If the model is a fair coin, this is pure randomness. If the model is two coins—one that always produces heads and one that always produces tails—there is no randomness. Speaking of the randomness of these outcomes necessitates reference to the data-generating process.

Suppose, arbitrarily, that our baseline prediction is that all outcomes are produced via a coin with probability $p_{i}^{0}=0.55$ of being heads. We first compute $A(p^{0};y)=0.53$ and then translate into an analogous coin of weight $w_{0}=0.67$ (see also Panel A in Fig 2). So as to forestall confusion, note the distinction between the implied coins, *w*_0_ and *w*_1_, and the coins with weights 0.5 and 0.95 used to generate the data; we make predictions about the latter and use the former to compute the IMV. Now, suppose that our enhanced prediction is $p_{i}^{1}=0.5$ for the first 20 observations (those produced by the fair coin) and $p_{i}^{1}=0.9$ for the second 20 observations (those produced by the weighted coin). We compute $A(p^{1};y)=0.63$ and translate that into $w_{1}=0.83$. Note that the coin suggested by *w*_1_ argues for a far less random system than the coin suggested by *w*_0_, this is intuitive given the fact that *p*^1^ is a far-superior approximation of the data-generating process. The improvement in prediction is now *ω* = 0 . 24, the IMV. The additional predictive information offered by the enhanced prediction translates to an expectation of winning nearly a quarter (i.e. 24 cents) for every dollar wagered.



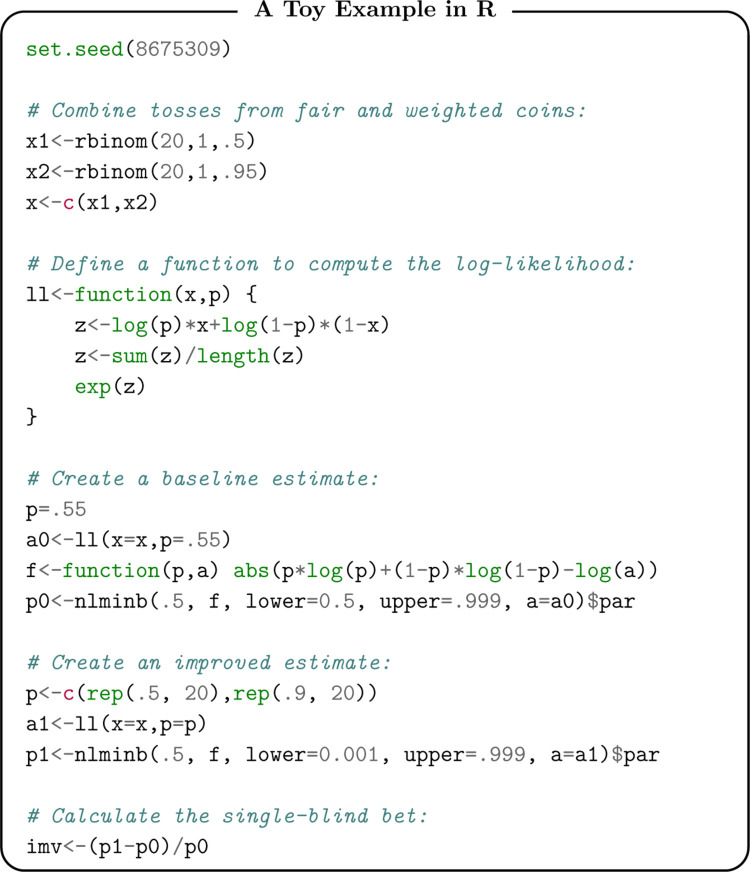



#### 2.3.2 Computation of the IMV in practice

The IMV can be computed directly with information about the likelihoods for the baseline and enhanced model; computation can be done via a simple application and functionality made available as part of the replication materials hosted on an organizational GitHub account (‘InterModelVigorish’). In practice, the quantity can be computed using in-sample or out-of-sample values for the likelihood (or, equivalently, based on training or test sets of estimated probabilities and observed responses). Training-based IMV estimates will be biased in favor of more complex models (see S1-III.4). Here, focus is on the prediction of test data given this problem and the generic issues associated with overfitting [20]. The key ideas related to out-of-sample prediction and cross validation are discussed at length elsewhere [20,21]. A note regarding computational costs: for a given set of predictions, the IMV can be computed very rapidly even for large datasets (i.e., the optimization in Eq 3 is fairly simple). However, producing out-of-sample predictions may be computationally complex; when used in our preferred out-of-sample framework, computational costs will thus depend on the complexity of implementing the predictive systems in question.

For values shown here, computation is typically done via a standard cross-validation procedure. We calculate the mean IMV based on 10-fold cross-validation: observations are randomly assigned into one of ten mutually exclusive folds with uniform probability (in a few cases, we use different numbers of folds or folding techniques where indicated in order to highlight the versatility of our approach). For a given fold, we first estimate model parameters using data assigned to other folds and then treat the given fold as an out-of-sample test dataset by computing *ω* using observations from this fold and predictions from the model constructed in the other folds. Uncertainty in the IMV can be assessed using standard deviations across the folds (see S1-III.3 for discussion regarding this as a way of describing uncertainty).

S1-II.2 describes an applied use case of the IMV; the Supporting Information accompanying this paper contains this example in R, but our online supporting repository of code also calculates this in Python and MATLAB. This example of how to use the IMV in practice takes the standard ‘Titanic’ dataset and predicts survival based on two simple logistic models: one that includes only a constant and one with sex and passenger class. In this context, these additional predictors are greatly predictive of survival (mean IMV is 0.352 with a standard deviation of 0.143) relative to prediction based on prevalence alone.

### 2.4 Properties of the IMV

Note several properties of the IMV. First, given that *A* is the geometric average likelihood for an observation, the IMV captures the expected winnings for the prediction of a single outcome generated by the enhanced coin. Second, note that the IMV is not symmetric. If interest is in the IMV associated with the side information in the baseline model relative to the enhanced model, the relevant quantity would be $\omega =\frac{w_{0}-w_{1}}{w_{1}}$. Third, observe that the IMV decreases as *w*_0_ increases for a fixed *w*_1_−*w*_0_; this behavior is shown in Panel B of Fig 2.

Is this desirable? Consider the house’s profit


$$\begin{array}{c} P(O)=\{\begin{array}{cc} \frac{1}{O}=\frac{1-w_{0}}{w_{0}}\; & \text{if the outcome is positive (with probability }w_{1}\text{)}\\ -1\; & \text{if the outcome is negative (with probability }1-w_{1}\text{)} \end{array} \end{array}$$ (7)

for a gamble based on a coin with $w_{1}=w(p^{1},y)$. *P* is a Bernoulli random variable whose support depends upon *O* but with parameter *w*_1_. If $O=\frac{w_{0}}{1-w_{0}}$, then $E(P)=\omega (p^{0},p^{1};y)=\frac{w_{1}-w_{0}}{w_{0}}$ (see Eq 5). Consider a potential alternative, $Z(P)=\frac{E(P)}{\sqrt{V(P)}}(\text{where}V$ is the variance operator). Given that *O* decreases as *w*_0_ nears unity, this would have the effect of our preferring gains, in terms of *w*_1_−*w*_0_, when *w*_0_ is near one (i.e. where the dashed curves in Panel B of Fig 2 are upward sloping). Doing so merely informs us that we are comparing changes in *w*_1_−*w*_0_ to very small levels of uncertainty. The goal in betting is to make money—to maximize *ω*—not to make smaller amounts of money in games with relatively little randomness; this logic applies to prediction of stochastic outcomes of scientific interest as well. Our metric produces values consistent with this logic. However, other approaches may require the prioritization of relatively small chanegs to *w*_1_−*w*_0_ when the underlying uncertainty is relatively low (e.g., something more akin to *Z* ( *P* ) ); our approach would be inappropriate in such cases.

S1-I contains additional arguments about the IMV. The IMV is a proper scoring rule [22] and is related to the “Kelly criterion” [23] which is an optimal approach to betting in certain scenarios that are related to the two bets considered in the construction of the IMV. Building on this connection to betting, we introduce comparisons to vigorishes from a number of common parlor games of chance (i.e., roulette, blackjack, and baccarat).

Finally, note the emphasis on predictions of unobserved (‘test’/‘out-of-sample’) data to compute the IMV. This is practically consequential given that, when used with training data, the IMV will always be biased in favor of more complex models. Our utilization of test data removes this bias—a fact which can be observed with a simple logistic regression example (S1-III.4)—but which is also consistent with the broader conceptual turn towards a focus on prediction [1]. In empirical cases, variation in the IMV across folds can be used as an index for sampling-related uncertainty in the IMV (see illustration in S1-III.3).

## 3 Simulation studies

We conduct several simulation studies pertaining to the IMV. These studies build on the simulations in earlier work which focused on a specific application of the IMV to the modeling of item responses [61]. We first describe specialized use cases that showcase the flexibility of the IMV given that it only relies upon predictions and outcomes and then describe a variety of simulation studies meant to contrast the IMV with common alternatives. We focus on a set of metrics—the AIC and BIC, pseudo-*R*^2^ approaches, the AUC, and the *F*_1_ score—that are meant to be representative of the large set of options available for use in this context. These metrics and the IMV behave similarly in many settings. In terms of the yes/no question “Does model A fit better than model B?”, we anticipate roughly similar answers from all the metrics; interest here is on scenarios that depict qualitative differences in the behavior of the magnitudes of these metrics.

### 3.1 Two specialized versions: The oracle and the overfit

The fact that the IMV is quite flexible and requires only fairly generic inputs—outcomes and two predictions—can be used to introduce two specialized versions of the IMV: the Oracle and Overfit. These are meant to help us better understand parameter recovery and overfitting in simulation studies. These metrics quantify the value of truth (when it is known) relative to estimates based on test data (Oracle) and training data (Overfit). We illustrate their use via a simple univariate logistic regression problem. We simulate data *y_i_* from a Bernoulli distribution based on


$$\begin{array}{c} p_{i}\equiv \mathrm{Pr}(y_{i}=1)=\sigma (\beta_{1}x_{i}) \end{array}$$ (8)

where $x_{i}∼\text{Normal}(0,1)\;\text{for}\;i\in \{1,...,N\}\;\text{and}\;\sigma$ is the logistic sigmoid, $\sigma (x)={\left(1+\exp(-x)\right)}^{-1}$. We vary *N* by sampling n∼Unif(log ⁡ 1050,log ⁡ 1010000) and setting *N*=10*^n^* and let $\beta_{1}\in \{0.01,0.1,0.5\}$. We estimate the logistic regression model using $\left(x_{i},y_{i}\right)$ from which we can create fitted values $\hat{p_{i}}$. We then generate a second set of outcomes, $y^{⋆}$, for the same values of $\beta_{1}\;\text{and}\;x$; that is, we use the same *p_i_* values to generate a second set of outcomes that will be used as out-of-sample test data.

Note that the models are trained on *y* while the $y^{⋆}$ outcomes are hypothetical test data (given that we use the same *x* to generate $y^{⋆}$ and *y*, the $\hat{p_{i}}$ are germane for each). For each simulated set of data, we then consider (using the order of arguments as in Eq 6):

- *ω*_0_: IMV(*ȳ*, $\hat{p_{i}}$; $y^{⋆})\;\text{where}\;ȳ=\frac{1}{N}\sum_{i}y_{i}$,
- Overfit: IMV($\hat{p_{i}}$, *p_i_*; *y*),
- Oracle: IMV($\hat{p_{i}}$, *p_i_*; $y^{⋆}$).

The oracle and overfit values are only available due to the fact that the data generating mechanism is known (i.e., we observe the true *p_i_*); while limited in scope they can be valuable in benchmarking the performance of estimates $\hat{p_{i}}$. The oracle and overfit values are computed based on the same probabilities, but with different data. Crucially, overfitting is indicated by negative values of the IMV. Negative overfit values imply that the estimates $\hat{p_{i}}$ are more valuable (because they are overfit to observed data) than the true *p_i_*. In contrast, the *ω*_0_ value shows that the IMV can function as a stand–alone metric since it relies only on $\hat{p_{i}}$ and the mean *ȳ* within the training data (i.e., the prevalence).

Results for 5,000 choices of *N* for each value of *β*_1_ are shown in Fig 3.1. Several key points emerge. When *β*_1_=0.01, there is (unsurprisingly) little value in using estimates to predict $y^{⋆}$ as opposed to just the mean (*ω*_0_ is near zero). However, there is a substantial cost paid due to overfitting for small *N*. For *β*_1_=0.1, note two key facts. First, the oracle IMV declines as sample size increases due to declines in $\left|p_{i}\;-\;\hat{p_{i}}\right|$ (i.e., estimates improve for large *N*). Second, the value associated with *ω*_0_ increases as a function of sample size for the same reason. For *β*_1_=0.5, a clearer influence of sample size on all three values is apparent. Values of *ω*_0_ increase as a function of sample size while both the oracle and overfit IMV values decline towards zero. This first simulation demonstrates that the IMV behaves sensibly in this simple context, and its flexibility allows us to utilize the Oracle and Overfit variants which may allow for future studies about the performance of various estimators.

**Fig 3:**
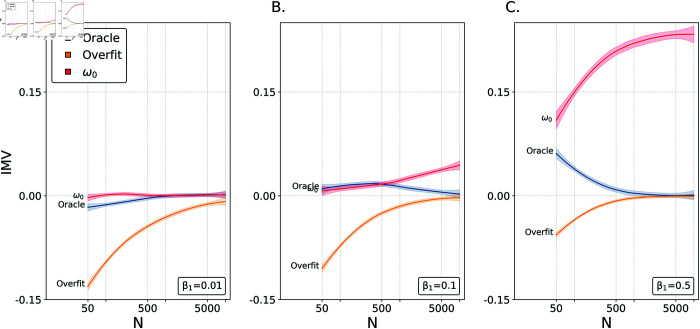
The Oracle, Overfit, and Omega. A comparison of *ω*_0_, oracle, and overfit values of IMV in the logistic regression context as a function of *β*_1_ and *N*, fit to 99.9% confidence intervals.

### 3.2 The IMV versus alternatives

#### 3.2.1 Comparisons to *R* ^2^,the AUC, and the *F*_1_score

We begin to contrast the IMV with alternatives by focusing on key measures of prediction accuracy: the *R*^2^, the *F*_1_ score, and the AUC. The below study is designed to emphasize the fundamental difference between the IMV and these alternatives (S1-III.1 contains a straightforward scenario wherein behavior across the indices is quite similar). We simulate data based on parameters ( *N* , *a* , *b* , *Ψ* , *ψ* ) . We generate *N* values from Beta (*a* , *b*) where we use the following density for the Beta distribution:


$$\begin{array}{c} f(x)=\Gamma (a+b)∕(\Gamma (a)\Gamma (b))x^{\left(a-1\right)}{\left(1-x\right)}^{\left(b-1\right)}. \end{array}$$ (9)

These are then linearly rescaled so as to lie on the interval ( *Ψ* , 1 − *Ψ* ) ; call these rescaled values *p*. Based on these true probabilities we generate two sets of outcomes (where the *i*–th outcome is generated via Bernoulli(*p_i_*)); the first set is used to derive a training mean *ȳ*, while the second set is treated as the test data. The *ψ* quantity is effectively error; we use *ψ* to generate an ‘estimate’ of *p*, denoted *p*_1_, where $p_{1}=p\pm \psi$ where we randomly choose to add or subtract. The parameter *ψ* serves as a proxy for the quality of estimates as $p_{1}\to p\;\text{as}\;\psi \to 0$. When *ψ* = 0 . 2, the difference |*p*_1_ − *p*| = 0.2 is large relative to the difference | *p* − *ȳ* | (i.e., for *a* = *b* = 1 we have *a* = *b* = 1 and thus if *Ψ* = 0 . 2 we have | *p* − *ȳ* | ≤ 0 . 3) meaning that *p*_1_ are low-quality estimates. We thus anticipate strong sensitivity in our metrics to the value of *ψ*.

**Fig 4:**
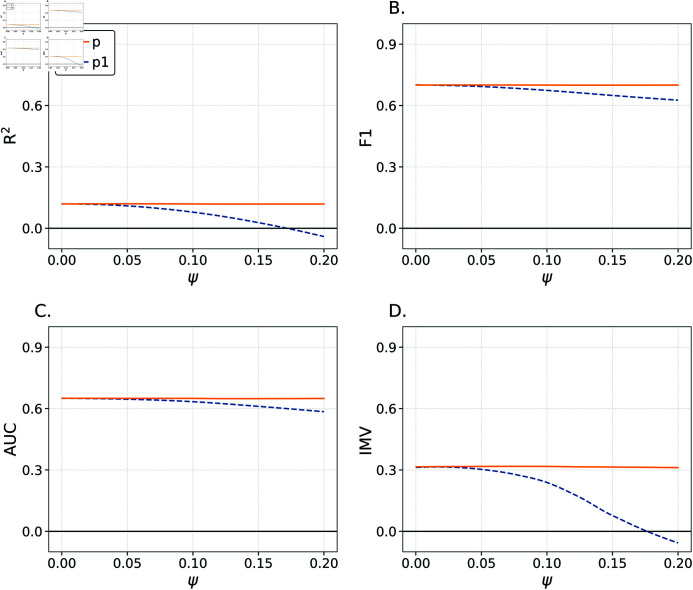
Metrics and *ψ.* Smoothed values of various metrics as a function of *ψ*.

We set *N* = 1000 , *Ψ* = 0 . 2 , *a* = 1 , *b* = 1 and consider 2,000 samples of *ψ* ∼ Unif ( 0 , *Ψ* ) . Results are shown in Fig 4 wherein we consider the metrics as a function of *ψ* (focusing on smoothed curves estimated via LOWESS regression of metrics on *ψ*). For each metric, we first compute the value when true *p* is known (e.g., for *R*^2^ we consider $1-\frac{\sum {\left(y_{i}-p_{i}\right)}^{2}}{\sum {\left(y_{i}-ȳ\right)}^{2}}$; we choose this approach given that it is used in [16]) as compared to prediction based on *ȳ*; these values are shown in the solid lines. Note that they are not dependent on *ψ*. We then compute each metric for *p*_1_ compared to *ȳ*; these are shown as dashed lines lines. As expected, we observe declines in the dashed lines as *ψ* increases. When *ψ* = 0 . 2 = *Ψ*, the *p*_1_ predictions are in fact worse than *ȳ* in predicting observations. We can illustrate this via a consideration of squared errors. We have ${\left(p_{1}-p\right)}^{2}=\psi^{2}=0.04$. Alternatively, we can compute $E({\left(ȳ-p\right)}^{2})=V(p)$. We can use the fact that *p* is a re-scaled Beta random variable to compute that


$$\begin{array}{c} V(p)=\frac{1}{12}{\left(1-2\Psi \right)}^{2}=0.03. \end{array}$$ (10)

Further, observe that the prediction errors should be equal in expectation when $\psi^{2}=\frac{{\left(1-2\Psi \right)}^{2}}{12}$ which has a solution for approximately *ψ*=0.17 which is where the dashed line crosses the origin for both *R*^2^ and the IMV. This fact is apparent for both the *R*^2^ and IMV which are below 0.

The AUC and *F*_1_ metrics are both fairly insensitive to *ψ*; the dashed lines decline from the solid lines, but the declines are relatively slight. From our perspective, these relatively slight changes—especially as *p*_1_ estimates become extremely low quality—make these metrics poor tools for a generalized understanding of the predictions from different models. While the R^2^ and IMV show sensitivity to *psi*, we turn now to a simulation study meant to illustrate their differences.

#### 3.2.2 A key distinction between the IMV and *R* ^2^

In Fig 4 both the IMV and *R*^2^ show relatively strong sensitivity to increased estimation error. However, they will not always be similar. We consider an analysis that contrasts the sensitivity of *R*^2^ to the strength of a predictor in a scenario wherein the prevalence of the outcome is being adjusted (via a logistic regression model) so as to make the IMV constant. We define $p_{i}=\text{Pr}(y_{i}=1)=\sigma (\beta_{0}\;+\;\beta_{1}x_{i})\;\text{for}\;x∼\text{Normal}(0,1)$. We then choose *β*_0_ values over a grid between 0 and 0.5 and we set *N* = 100 , 000 to make clear that our results aren’t driven by sample size. We specify a value for *ω* ∈ { 0 . 01 , 0 . 1 } that determines the desired IMV for prediction relative to the mean (i.e., *ω*_0_ as in the discussion of the oracle and the overfit indices). Our goal is to then find the necessary *β*_1_ to generate the specified *ω* value; we are effectively querying the trade-off in *β*_1_ necessary to maintain a constant IMV when we increase the prevalence via *β*_0_. As *β*_0_ increases, a larger *β*_1_ value is required to hold *ω* constant. We identify the appropriate *β*_1_ (using an optimizer) and then use these values to simulate data ( *x* , *y* ) from which we construct $R^{2}=1\;-\;\sum {\left(y_{i}\;-\;p_{i}\right)}^{2}∕\sum {\left(y_{i}\;-\;\sigma (\beta_{0})\right)}^{2}$ (we use the true *p_i_* here so as to make clear that this is not a point about estimation error).

**Fig 5:**
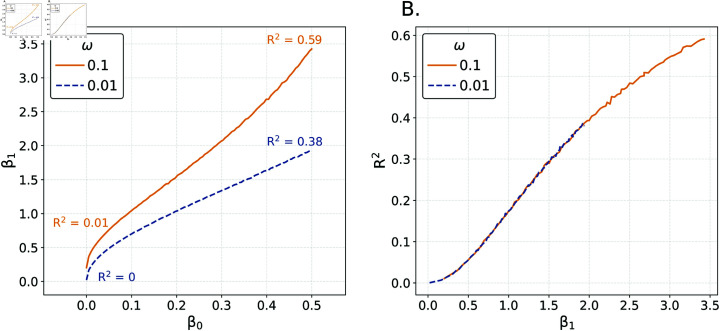
Comparisons of prevalence. Comparisons of prevalence versus *β*_0_ and *β*_1_ for constant values of *ω*.

Fig 5 examines the (*β*_0_,*β*_1_) curves while also providing information about the *R*^2^ values for extreme choices of *β*_0_ (left panel). Each point on a given line has the same IMV. For a given value of *β*_0_, the solid line is above the dashed line. This is due to the fact that from the perspective of the IMV, an increase in *β*_0_ decreases randomness in the outcomes and therefore larger values of *β*_1_ are necessary to generate the same predictive value. We then compare *β*_1_ and the resulting *R*^2^ (right panel). As one would expect, an increase in *β*_1_ leads to an increase in *R*^2^. However, the two lines representing the different choices of *ω* are overlapping. The *R*^2^ value is highly sensitive to *β*_1_ while the IMV is sensitive to both *β*_1_ and *β*_0_. This divergence between the meaning of *ω* and *R*^2^ is crucial in explicating the novel information provided by the IMV (similar evidence regarding the relationship between these parameters and prevalence can also be found in S1-III.2).

#### 3.2.3 The IMV versus information criteria

We now consider the AIC and BIC [7] vis-a-vis the IMV in a simulation study (while there are similarities between the AIC and BIC, note that there are also important differences between them [24]). These quantities are based on adjustments to the likelihood given the number of estimated parameters. These adjustments are meant to minimize overfitting; i.e., the AIC is asymptotically equivalent to leave-one-out cross validation [25] (although conventional usage of the AIC may be sub-optimal if the underlying statistical model is mis-specified [26]). Given modern computational power, such adjustments can perhaps be replaced in favor of the testing of different models in novel data not used for training (i.e., model estimation). While adjustments are available to remove dependency on sample size [27], information criteria values are also sample-size dependent; this dependence makes generalizations challenging, a point made in initial simulation studies with the IMV [61].

**Fig 6:**
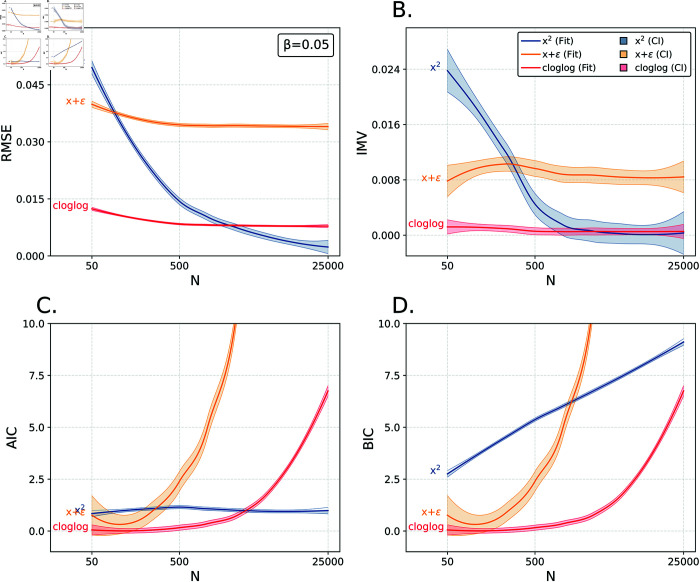
Oracle, Overfit, and Omega. Comparison of IMV and information criteria for different choices of *N*. Columns represent IMV, AIC, and BIC from left to right respectively. Y-axes are truncated so as to emhasize area of key variation.

To illustrate the substantive differences between the AIC/BIC and the IMV, we consider the simulation study summarized in Fig 6. For a choice of ( *N* , *β* ) , we generate *N* values *y* based on * Pr* ⁡ ( *y* = 1 | *x* ) = *σ* ( *βx* ) . We then fit the correct model and contrast it with three alternatives: (1) an overfit model with quadratic terms in *x*, (2) a model where we observe *x* rather than *x* (where ε ∼ Normal(0,0.3^2^)), and (3) a model where the complementary log-log link is used instead of the logistic. We fix *β* = 0 . 5 and vary *N* (we sample n∼Unif(log ⁡ 1050,log ⁡ 1025000) and let *N*=10*^n^*) so as to focus on the behavior of these curves in the context of changing sample size. We generate two sets of outcomes; the first is used for estimation, and the second for computation of out-of-sample IMV values. Each curve is based on a comparison of the correct model to one of the incorrectly specified alternatives. To supplement our interpretation of these curves, we also add the root-mean-square-error of the estimated probabilities as compared to true probabilities.

Fig 6 readily captures the difference in behavior across the IMV and the information criteria. Consider the comparison of the overfit model that includes the quadratic term of the true model (the blue curve). The difference in prediction error (panel A) declines to zero as *N* increases; the IMV (panel B) behaves similarly. The IMV curve captures the critical information: with small *N*, an overfit model can be costly but an overfit model with large *N* behaves functionally equivalently to the true model. In contrast, the AIC (panel C) is constant as a function of sample size; in fact, the AIC is roughly unity for all *N* (as expected given that the difference between the models is the estimation of a single additional parameter). The BIC (panel D) increases as a function of sample size.

For the yellow curves—which compare the prediction based on a noisy covariate compared to the true model—there is a relative insensitivity in the IMV value to sample size; this is to be expected given that the attenuation bias does not depend on sample size (see Eq 26.8 in [28]) and is confirmed by the RMSE. The information criteria are, on the other hand, strongly increasing as a function of sample size with respect to this mis-specification. Finally, the mis-specified link (red curve) has a consistently small IMV with some decline as *N* increases; again, the RMSE behaves similarly. In contrast, the information criteria sharply differentiates between the two models for large *N*. Our aim is not to critique the information criteria; these metrics behave appropriately given their designed purpose. But, clearly, the IMV displays distinctive behavior that quantifies the difference between predictions (rather than attempt to authoritatively arbitrate in favor of one set of predictions, as with the information criteria) and this behavior matches that of the prediction error as a function of sample size.

### 3.3 Summary of simulation results

The simulation studies described here indicate some unique features of the IMV relative to alternatives. It is clearly distinguished from metrics like the AIC and BIC in that its variation as a function of sample size only matters to the extent that sample size improves prediction (rather than allowing for very precise adjudication between fairly similar approaches). This evidence cumulatively suggests that the IMV offers novel perspectives on fit when considering binary outcomes. Again, note that the IMV behaves similar to other metrics if interest hinges on a choice between two models. However, the IMV is designed to be portable such that values can be compared in a straightforward way across settings. That is, the IMV can be used to not just choose between models, but to also understand how much better one model is than another in a consistent manner. Further, the flexibility of the IMV allows for easy computation of the Oracle and Overfit quantities. These quantities can be used to, for example, study the implications of estimation error.

## 4 Empirical illustrations

We now consider the IMV’s potential use in a range of canonical examples across the social sciences. These examples are meant to offer useful benchmarks in the form of IMV values against which future work can be compared, and to illustrate the fact that the IMV can be interpreted as a meangingful quantity even when there are variations in prevalence. Focus is on the IMV rather than alternative metrics. In our view, comparisons to alternatives are best made via controlled simulation settings (as above); this omission is not meant to imply that alternative metrics fail to offer complementary information.

### 4.1 Illustration One: Prediction of health outcomes

Predictive models are being used to study a variety of health-related phenomena including the social determinants of health [29] and age-related social care [30]. To illustrate how the IMV can be used to index such predictions, we build models related to health outcomes using data from a population-based survey; the Health and Retirement Study (HRS [31,32]; see S1-IV.1 for further details). We illustrate how the IMV can be used to quantify prediction of health outcomes. The risk of these health outcomes varies substantially as respondents age (see S1–S6 Figs). We thus focus on prediction within age bins. However, given the change in prevalence as a function of age it would be challenging to examine changes in age-related predictability of these health risks using metrics that do not appropriately account for changes in prevalence; the IMV is thus a useful tool for this exercise.

Consider first predictions of health outcomes based solely on demographic information. Certain outcomes—high blood pressure and arthritis in relatively young respondents, and heart disease in relatively old respondents—are predicted with *ω* > 0 . 02 using race and sex relative to prevalence alone while others (e.g., stroke, death) are predicted more weakly (results shown in S1–S7 Figs). While we focus on predictions of health problems in age bins, we can utilize predictions of health as a function of age as a benchmark; age is maximally predictive of heart disease (*ω* = 0 . 016) compared to prevalence alone. We next consider prediction based on adding educational attainment. Relative to prediction using demographics, gains from adding information on education are extremely modest with *ω* < 0 . 01 in virtually all cases. This limited increase in prediction associated with inclusion of information about education is noteworthy given substantial interest in educational disparities in health conditions [33].

We next consider predictions based on relatively expensive-to-collect pieces of health data: cognition and physical functioning (as measured by grip and gait). These expensive data are, for certain outcomes, as predictive as information about respondent age. Amongst older respondents, the cognitive score predicts death and proxy-based responding (*ω* ≈ 0 . 02) at the next wave. Turning to grip and gait, they predict, for example, heart disease amongst respondents aged 80 (*ω* = 0 . 019). As a contrast, we can compare these predictions with those from clinical samples. Prediction of health outcomes in clinical samples is far superior (see Table 1): e.g., heart disease *ω* = 0 . 12, Breast Cancer *ω* = 0 . 53, diabetes *ω* = 0 . 62. These differences presumably reflect the value of predictors ascertainable in clinic settings and show the relatively limited value of similar covariates designed to be informative about individual health in population studies (i.e., grip and gait).

### 4.2 Illustration Two: Prediction of political party affiliation

**Fig 7:**
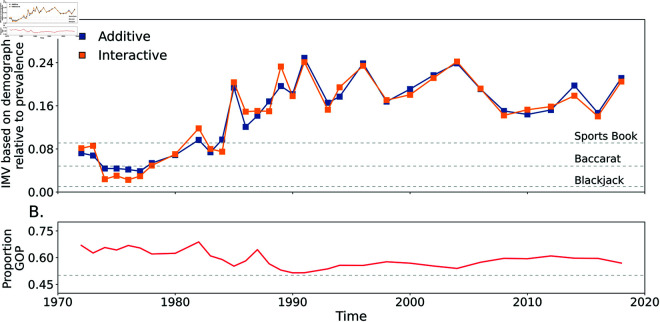
IMV and the GSS. Panel A (top) shows the IMV for prediction of political party affiliation across GSS survey years and Panel B (bottom) the proportion of GOP (“Grand Old Party”) within the GSS respondents by year.

Predicting political orientation has recently become a mainstay within the field of social data science [34], with voter-based microtargeting (for the purpose of political messaging) allegedly occurring regularly and in high-profile, consequential circumstances [35]. We examine the relative information content held within simple demographic variables by predicting political party affiliation using data from the General Social Survey (GSS, [36]; detail in S1-IV.2). The relative popularity of the political parties has also changed over time (see Fig 7 Panel B) thus making it challenging to quantify temporal changes in the degree to which party affiliation is structured by these demographic features using many common metrics. The IMV is well-suited to this problem.

We predict affiliation using age, sex, and race. Fig 7 Panel A shows the IMV of demographics in predicting party affiliation beginning in 1970. There is a sharp increase across the 1980s in the predictive power of demographics. After a peak in the 1990s near *ω* = 0 . 25, the predictive power declines to roughly between 0.15 and 0.2 between 2000 and 2020. There are only minor differences between additive and interactive models. Especially in the 1990s, party affiliation is relatively strongly predicted by demographic features; the IMV is roughly an order of magnitude higher than those observed with health outcomes in the HRS. Our results complement others discussing the changing nature of US political partisanship [37,38] and suggest a potentially strikingly high level of predictability of partisan affiliation as a function of demographics, not least before ‘big’ data [39] or psychological profiles are integrated into the domain.

### 4.3 Illustration Three: The Fragile Families Challenge

The Fragile Families Challenge (FFC; [16]; detail in S1-IV.3) aimed to quantify the level of predictability in sociological and behavioural life course outcomes using data from Fragile Families and Child and Wellbeing Study (FFCWS; [40]). Widely heralded as a (much-needed and) progressive approach to bringing out-of-sample prediction into the main-stream social science literature [9,10,41], it incorporated a ‘common task method’ [42] where 160 teams of independent researchers submitted predictions based on a reserved, unseen hold-out set of six key outcomes in Wave Six of the FFCWS (three of which were binary). Teams differed substantially in the methodological sophistication of their submitted approaches (see also [43]). Some built models based on theory and prior research [44], whereas others began with many variables [45] or took a ‘human in the loop’ based approach [46].

**Fig 8:**
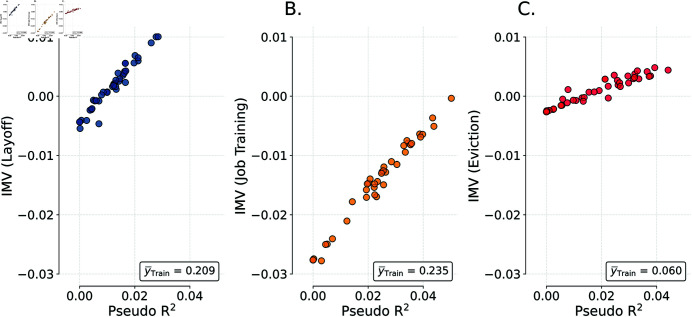
Re-evaluating the Fragile Families Challenge. IMV metrics (from each submission with a Pseudo R^2^>0) plotted against the Pseudo R^2^ for all FFC submissions when evaluated against a four variable benchmark [16]. Panels A-C evaluate Layoff, Job Training and Eviction respectively.

The binary outcomes had different prevalences in the training data (21%, 23%, and 6% for layoff, job training, and eviction respectively); these differences are a first challenge in making comparisons between the *R*^2^ values used in the original paper (i.e., Brier Skill Scores) which are difficult to interpret across outcomes. Further, the baseline models were differentially successful in predicting outcomes, thus furthering the challenge of making comparisons regarding the degree to which sophisticated approaches led to improved prediction. The IMV also allows us to unequivocally state that the benefits of the ‘enhanced’ models submitted by challenge participants were unilaterally small. To emphasize the IMV’s utility, consider a simple question: for which outcome were predictive gains the largest? Mirroring the FFC, we can consider this using *R*^2^ values (See Fig 8). We first compute the *R*^2^ value based on the benchmark model (0.009, 0.049, 0.014 for layoffs, job training, and evictions respectively) and then compute it for each candidate model and look at the difference in these quantities. For the three outcomes, the maximal *R*^2^ values were 0.028, 0.050, and 0.044 respectively. Taking differences yields 0.019, 0.0004, and 0.030; from this perspective, the modeling innovations were most useful in improving predictions of evictions and layoffs with the biggest gain seeming to come from predicting evictions.

Alternatively, we can use the IMV to analyze this question. In terms of rank-ordering, the IMV and the *R*^2^ values provide similar information (the Spearman correlations between these are greater than 0.97 for all three outcomes); there is little difference across the two metrics as to the rank-ordering of the modeling approaches in terms of the relative improvements. We again observe that the IMV and a metric such as the *R*^2^ will produce similar information if there is interest in making a simple yes/no decision related to any single outcome. However, the IMV offers a different perspective on the question of where the gains are the most impressive across outcomes. Across layoffs and eviction, maximal IMVs (between FFC submissions and the benchmark model) are 0.010 and 0.005 (excluding job training, which was zero to four decimal places). The IMV indicates that the reduction in uncertainty provided by the best FFC model is twice as valuable in removing uncertainty when predicting layoffs as the best FFC model for predicting evictions. The ordering as compared to the *R*^2^ approach is reversed and, more critically, we can meaningfully compare the magnitudes of the IMV given that they are designed to be used in such a manner.

### 4.4 Empirical benchmarks

**Table 1:** Summary of results. IMV for various empirical illustrations (alongside gambling comparators) plus prevalences; results ordered by IMV. The IMV is the mean *ω* across many folds (along with the SD of the *ω* so as to indicate uncertainty). In some examples (e.g. FFC re-examination) there is only one fold. In such cases (and in casino games), no SD is reported.

Binary Outcome	Data	Model 1	Model 2	Prevalence	Mean *ω_k_*	SD $\omega_{k}^{†}$
Job Training	FFC	Benchmark Model	Top predictor	0.23	0.0000	–
Math item responses	PISA	2PL	3PL	0.47	0.002	3e-4
Eviction	FFC	Benchmark Model	Top predictor	0.06	0.005	–
Eviction	FFC	${ȳ}_{\text{Train}}$	Top predictor	0.06	0.007	–
High blood pressure (age 63)	HRS	Age and sex	+ education	0.52	0.008	0.017
*Blackjack*					*0.010*
Survival on Titanic	Titanic	Logistic Regression	LightGBM	0.38	0.010	0.048
Math item responses	PISA	Rasch	2PL	0.47	0.010	0.001
Layoff	FFC	Benchmark Model	Top predictor	0.21	0.010	–
Layoff	FFC	${ȳ}_{\text{Train}}$	Top predictor	0.21	0.014	–
*Knowledge of initial coin state*					*0.019*
Death (age 90)	HRS	Age, sex, and education	+ cognition	0.29	0.024	0.022
Heart problems (age 80)	HRS	Age, sex, and education	+ grip and gait	0.39	0.026	0.063
Political Party affiliation (1976)	GSS	Prevalence	GLM based on age, sex and race	0.66	0.026	0.037
Job training	FFC	${ȳ}_{\text{Train}}$	Top predictor	0.23	0.028	–
*Baccarat*					*0.048*
High blood pressure (age 63)	HRS	Prevalence	Age and sex	0.52	0.067	0.108
High family income	[47]	SAT scores	+ topics	0.50	0.073	0.006
*Sports book*					*0.091*
COVID Infection	[48]	Prevalence	First month, small spec GLM	0.31	0.092	0.098
Heart disease	[49]	Prevalence	GLM	0.28	0.123	0.287
Survival on Titanic	Titanic	Prevalence	+ Sex + ticket class	0.38	0.352	0.143
Home win in European football (average)	[50]	Prevalence	Network model	0.64	0.159	–
Nonmarine coarse siltstone	[51]	Prevalence	GLM	0.23	0.163	0.033
Skin ID	[52]	Prevalence	GLM	0.79	0.196	0.004
Hospital re-admissions in DM patients	[53]	Prevalence	GLM	0.46	0.196	0.015
Excess alcohol consumption	[54]	Prevalence	GLM	0.51	0.245	0.170
Political Party affiliation (1991)	GSS	Prevalence	GLM based on age, sex, and race	0.51	0.256	0.094
Glass Manufacturing process	[55]	Prevalence	GLM	0.41	0.420	0.078
Marine siltstone and shale (v. Mudstone)	[51]	Prevalence	GLM	0.46	0.446	0.157
Breast Cancer	[56]	Prevalence	GLM	0.37	0.526	0.157
Early detection of diabetes	[57]	Prevalence	GLM	0.62	0.617	0.230
Abalone rings	[58]	Prevalence	GLM	0.50	0.667	0.031

FFC results based on the top-performing model. Predictions of health status from HRS selected by identifying the maximum IMV for each pair of model contrasts. For the GSS application, max and min values for additive models across survey years are shown. For games of chance, the house vigorish is shown.

^†^ Values omitted when the IMV is based on out-of-sample prediction from a single test dataset.

Alongside the core empirical illustrations, additional examples further demonstrate the range of IMV values. The first is the prediction of item responses to cognitive tasks using item response theory models using data from the OECD’s Programme for International Student Assessment (PISA; [59]). Another is the prediction of social class using natural language processing, based on recent work using text data from college application essays [47]. We also consider whether a home team wins in European football [50], and the prediction of a positive COVID diagnosis based on symptomology [48] (see S1-V.2). Finally, we consider the prediction of outcomes from a variety of scientific disciplines (e.g., biology, physics, medicine; [55]) that serve as interesting contrasts of more highly predictive systems. See S1-V for additional detail on these data.

Table 1 contains IMV values observed from prediction exercises across all aforementioned domains. We can compare these results to the vigorish benchmarks from popular games of chance as well as the profit associated with knowledge of the coin’s initial state (i.e., head up?) pre-toss [60]. As one example of a relatively weak improvement in predictive value, increasing model complexity for item responses (S1-V.3) on cognitive assessments for adolescents adds limited predictive value, *ω* ≤ 0 . 01 (these values are similar to those observed for the FFC predictions). Concerning an NLP example: the text used in college admissions essays predicted whether an applicant’s family income was above or below the median with *ω* = 0 . 073 (S1-V.4). We also replicated previous observations [50] of a changing patterns of predictability of a home team victory in European football over time (See S1-V.5), which are surprisingly predictable (*ω* = 0 . 159). Turning to examples from the physical and biological sciences, there are numerous cases (e.g., prediction of abalone rings, *ω* = 0 . 667, or glass type, *ω* = 0 . 420, see S1-V.6) that serve to benchmark the high levels of predictive value associated with simple models for outcomes determined by well-understood scientific processes. These predictions are, in many cases, orders of magnitudes more valuable than those based on, for example, predictions of health problems in population-based surveys.

Table 1 emphasizes the flexibility of the IMV in allowing for straightforward comparisons across outcomes irrespective of prevalence or modeling strategy. It also provides a range of values against which future studies can be benchmarked. However, we deliberately avoid suggesting specific values as benchmarks. The fact that the IMV can be interpreted in monetary terms suggests that the answer to “What is a large IMV?” is as context-dependent as the answer to “What is a large amount of money?”. Future work can place IMVs for a given scenario in a range of contexts using results from Table 1 which includes a large number of outcomes that are both highly stochastic (in the context of predictions made here), such as evictions and layoffs, as well as relatively strongly determined, such as the relationship between age and rings amongst abalones.

## 5 Discussion

As our capacities for computation and data collection expand, the applicability and relevance of prediction increases. Therefore, so does our need to evaluate prediction in a consistent and tractable fashion. The IMV is a flexible and portable metric for evaluating predictive accuracy with binary outcomes. Our approach focuses on anchoring a given predictive system to a physical analogue with readily understood statistical properties: weighted coins. The coins establish a system that informs us about the expected winnings associated with an improvement in prediction. We compare this approach via simulation to alternative metrics of predictive accuracy and then undertake various simulated and empirical illustrations. Note that the IMV is portable—values can be consistently interpreted across outcomes—and thus can be used across a large range of scientific outcomes and predictive models.

We emphasize a few select facts about the IMV that are intriguing arguments for its use in future settings. First, simulation studies suggest that the IMV is quite sensitive to error in estimated probabilities (i.e., Fig 4) and is also distinct from other metrics that are similarly sensitive in terms of how they respond to changes in prevalence (i.e., Fig 5). Second, the fact that the IMV is inherently a metric of change allows it to be used in interesting ways; in particular, the Oracle and Overfit metrics might be used in simulation work to further our understanding of estimation error, sample size, and the problem of overfitting in many scenarios. Third, the IMV can be used to clarify the meaning of logistic regression coefficients (see S1-V.1.1). Understanding of those coefficients is frequently challenging given that they require discussion of odds ratios; the IMV can be used to straightforwardly state the relative predictive value of a given covariate in such models in ways that might help ease understanding in future work. Note also that conventional metrics are not helpful for clarifying the relative predictive contribution of a predictor in a portable fashion.

One clear distinguishing feature of the IMV is its sensitivity to prevalence. As an outcome’s prevalence moves away from 0.5, this leads to an increase in *w*_0_; thus, a given value of *w*_1_−*w*_0_ will be associated with a smaller IMV as prevalence increases. When discussing the proprties of the IMV, we discussed this behavior based on a consideration of profit maximization in gambling. This defense does not require one to think of prediction as gambling but merely emphasizes that highly prevalent outcomes have less uncertainty as compared to less prevalent ones. This fact is critical in understanding why the IMV behaves as it does. Here we offer an alternative rationale. Increases in *w*_0_ can occur due to either increases in prevalence or increases in the predictive capacity of the baseline model. Focusing on the latter, the IMV’s behavior is consistent with the logic that increases in predictive power (i.e., values of *w*_1_−*w*_0_) are more meaningful in terms of resolving uncertainty when we have less predictive power from the baseline model (i.e., a smaller *w*_0_). In our view, this logic is persuasive given that predictive innovations for outcomes that are poorly understood are harder to come by relative to further increasing clarity about relatively well-understood outcomes.

We offer a wide range of empirical illustrations to showcase the IMV. These examples illustrate a wide range of predictability—over two orders of magnitude—of outcomes across a range of scientific disciplines. While we refrain from offering specific values to which future IMVs can be compared, the range shown in Table 1 will allow for rapid contextualization of future results. We also use the IMV to illustrate change in prediction over time. The study of party affiliation in the GSS shows how the metric can be used to index changes in the predictability of outcomes over time. Past approaches may have documented changes in the level of a covariate’s estimated magnitude over time but interpretation of such estimates is compromised if the prevalence is fluctuating as it clearly is here; the IMV resolves this issue. The IMV can also be used to clarify interpretation and comparison of logistic regression coefficients (e.g., see discussion of the role of sex in predicting death in the Titanic disaster in S1-V.1).

Alongside the different empirical settings, the illustrations also make use of a wide range of modeling approaches. Alongside logistic regression examples, we also make use of latent variable models (i.e. IRT in the context of PISA; see also [61]), machine learning approaches (in the FFC), and natural language processing (essays and income). The IMV represents a reasonable evaluation metric for endeavours such as Kaggle-like competitions (S1-V.1.2) and is flexible in terms of its ability to allow for comparisons of different specifications and estimators given that it only requires outcomes and associated predictions.

The IMV can be used to compare a multitude of outcomes, but such comparisons need to allow for the role of context. For example, a small increase in an already highly predictive medical diagnostic test may have major implications in terms of time, money, and human lives that render such gains much more important than similar increases in other settings. The portability of the IMV values allows for ready comparisons, but these comparisons will need to be informed by other concerns. A related limitation of the IMV is that it does not differentially weight false positives and negatives. It may need to be used—as with other probabilistic loss functions—with care in settings wherein there is interest in minimizing one of those two quantities. Future work could focus on extending the IMV to incorporate the broader notion of cost/loss or utility functions used in decision theory [62].

Scientists have long prioritized knowledge about *what* predicts an outcome. Interest, however, is turning towards *how* predictable an outcome is (e.g., [16]) and specifically to decomposing ‘predictability’ in social systems and life prediction tasks [63]. Having metrics that can be readily used to understand the degree of randomness in a given predictive system is thus highly desirable. The metric introduced here is relatively easy to compute—based on an intuitive analogy to a physical system—and has a range of desirable properties. The scientific community has accumulated a multitude of insights regarding what factors may be relevant for predicting certain outcomes; our work is meant to offer a tool for further advancing our understanding of the stochastic nature of those outcomes.

## Supporting information

S1 File Supporting information for The InterModel Vigorish (IMV) as a flexible and portable approach for quantifying predictive accuracy with binary outcomes. (PDF)
