# Supplementary material for: The InterModel Vigorish (IMV) as a flexible and portable approach for quantifying predictive accuracy with binary outcomes
Source: PLoS One. 2025 Mar 21;20(3):e0316491. doi: 10.1371/journal.pone.0316491 (PMC11927910; doi:10.1371/journal.pone.0316491)
Supplement: S1 File — (PDF) [file pone.0316491.s001.pdf]

S1. Supporting Information for The InterModel Vigorish (IMV) as a flexible and portable approach for quantifying predictive accuracy with binary outcomes

Contents

I Additional Notes on the IMV **2**
- I.1 The IMV as a proper scoring rule . . . 2
- I.2 Connections to betting and the Kelly Criterion . . . 2

II Practical examples of IMV calculation **4**
- II.1 The Toy Example (Section 2.3.1) in different languages . . . 4
- II.2 A fully worked example . . . 7

III Additional Simulation studies **9**
- III.1 The IMV versus Alternative Metrics . . . 9
- III.2 Fit statistics as a function of prevalence . . . 9
- III.3 Variation in the IMV across folds . . . 10
- III.4 The IMV and overfitting . . . 10

IV Additional Details on Core Empirical Illustrations **15**
- IV.1 The Health and Retirement Study . . . 15
- IV.2 The General Social Survey . . . 15
- IV.3 The Fragile Families Challenge . . . 16

V Additional Empirical Examples **18**
- V.1 Survival on the Titanic . . . 18
  - V.1.1 Logistic Regression Estimates . . . 18
  - V.1.2 Prediction Competitions . . . 19
- V.2 The COVID-19 Symptom Study . . . 20
- V.3 OECD's Programme for International Student Assessment (PISA) . . . 21
- V.4 Predicting family income from text data . . . 21
- V.5 Predicting whether home team wins football in professional European competitions . . . 22
- V.6 Raw prediction examples . . . 22

I Additional Notes on the IMV

I.1 The IMV as a proper scoring rule

In the realm of probabilistic forecasting, scoring rules are used to evaluate the quality of predictions [1]. As the IMV requires probabilities as inputs, it makes sense to consider it as a potential scoring rule. Recall that the IMV is meant to quantify the predictive gain of one model relative to another. Despite this difference in application, if we fix the baseline model, the IMV constitutes a scoring rule for the evaluation of the predictive quality of enhanced models. A desirable property of scoring rules is that they are *strictly proper*, or they produce a maximum expected score if and only if the forecasted probabilities are equal to the true probabilities [1]. As the IMV with its fixed baseline model is a re-scaling of the log likelihood of the enhanced model, and as the log likelihood itself is a proper scoring rule (the *logarithmic score*), we see that the IMV with fixed baseline model is itself a strictly proper scoring rule. As such, it prioritizes accurately describing the underlying probability distribution which generates the observed data over attributing extreme confidence to predictions.

I.2 Connections to betting and the Kelly Criterion

The interpretation of the IMV in non-statistical terms (given that it calculates the expected payoff from an uncertain investment) can be immediately connected to expected returns in other domains outside of formal statistical modelling. For example, our approach shares some of the logic in previous derivations of key principles of decision analysis from the basis of coin-tossing [2]. An even more useful comparison is to gambling. The vigorish (the "profit" associated with taking the bet that is generated by assignment of asymmetric odds to competing outcomes) at most sports books is around 10% [3] implying that $\omega = 0.091$. This is relatively high; vigorishes for baccarat and blackjack (under certain assumptions, such as play being strategically optimal) are 5% [4] and 1% [5] respectively, which translate into expected winnings of 0.048 and 0.0099 cents per dollar respectively for the casino.[1] We use these as benchmarks in empirical illustrations below.

Our construction of the IMV is also related to one approach to betting: the "Kelly Criterion" [6]. This relates to an optimal strategy for uncertain investment. Suppose that in an (infinite) series of instances one is faced with a bet that pays out V dollars for each dollar wagered in the event of a win, while one loses the whole wager in the event of a loss. If the probability of winning is p, what proportion of one's wealth should they bet at each instance? The Kelly criterion proposes that one can maximize long-run wealth via optimal wagering. In particular, the optimal proportion of one's wealth one should wager (the Kelly bet) is

$$K \equiv p - \frac{1-p}{V}. \tag{S1}$$

In our case, the amount won (per dollar) in the event of a win is the one implied by the benchmark model, i.e., $V = 1/O_0 = (1-w_0)/w_0$, while the probability of winning (for the player who uses the enhanced model) is $p = w_1$. The Kelly bet is, then,

$$K = w_1 - \frac{1-w_1}{\frac{1-w_0}{w_0}} = \frac{w_1(1-w_0) - w_0(1-w_1)}{1-w_0} = \frac{w_1 - w_0}{1-w_0}. \tag{S2}$$

Notice the similarity between the Kelly bet (Eqn. S1) and the IMV (Eqn. 11). For example, if you wanted to consider the vigorish from the perspective of the amount won per dollar wagered by the gambler, you would essentially use the Kelly criterion. The numerator in both formulas is $w_1 - w_0$, but the denominator differs. The values of both metrics increase in w_1 and decrease in w_0 if $w_1 < 1$. Suppose, however, that the better model is perfect at predicting the outcomes (i.e., $w_1 = 1$). This implies $K = 1$ which is is intuitive: if one is certain to win the wager, one should bet their entire bankroll. This can be seen in the contour plots for IMV and the Kelly bet in Figure S1; note the flat line associated with $w_1 = 1$. From our perspective,

[1] Consider a hypothetical two-outcome betting example where a book prices the ('European'/decimal) odds of an event happening at 1.5 (i.e. net 50 cents profit on a dollar successfully wagered), and offers odds on the same event not happening at 2.5. The 'over-round' (O) on this book is calculated as the sum of the implied probabilities: $(\frac{1}{1.5} + \frac{1}{2.5}) = 106.67\%$, and the 'vig' as $\omega = \frac{O-100}{O} = 0.0625$.

Figure S1: Contour plots for the IMV and the Kelly bet given w_0 and w_1.

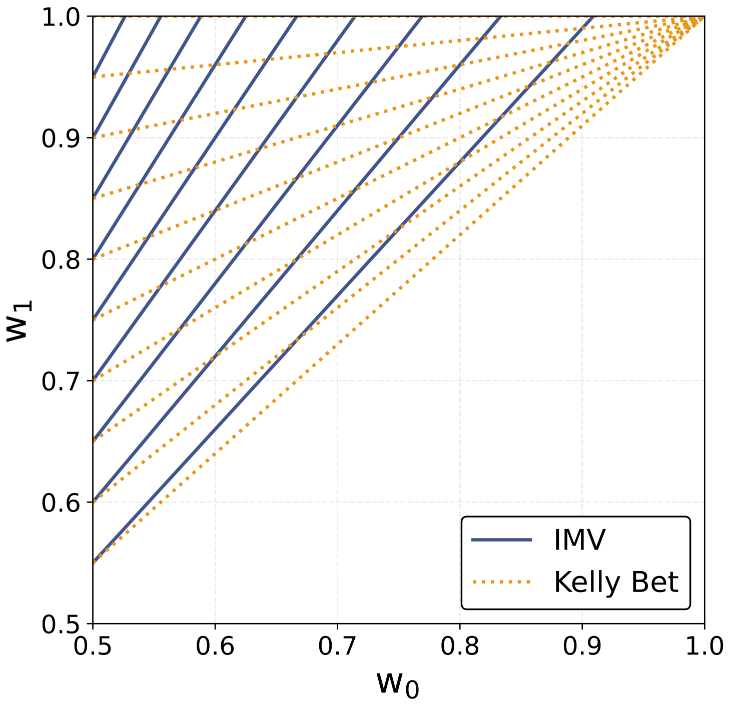

the lack of discrimination by K between values of w_0 when $w_1 = 1$ suggests a limited utility of K for model comparisons (admittedly in an edge case) given that there is much more to be gained when w_0 is relatively small. If $w_0 = 0.999$, one should bet their bankroll, but accruing wealth will be a slow process.

II Practical examples of IMV calculation

II.1 The Toy Example (Section 2.3.1) in different languages

We now offer additional snippets of code which illustrate how the IMV can be calculated in the context of the 'toy example' in Section 2.3.1 of the main body of text. We do this first in Python, and then in MATLAB. The three examples provide IMV values equivalent to at least six decimal places of procession (0.237229), despite the fact that three different optimisation functions are used.

A Toy Example in Python

```
import numpy as np
from scipy.optimize import minimize


def ll(x, p):
    """Calculate the log-likelihood"""
    z = np.log(p) * x + np.log(1 - p) * (1 - x)
    return np.exp(np.sum(z) / len(z))


def get_w(a, guess=0.5, bounds=[(0.001, 0.999)]):
    """Calculate 'w' using optimization"""
    res = minimize(minimize_me, guess, args=a,
                   options={'ftol': 0, 'gtol': 1e-09},
                   method='L-BFGS-B', bounds=bounds)
    return res.x[0]


def minimize_me(p, a):
    """Function to be minimized"""
    return abs(p * np.log(p) + (1 - p) * np.log(1 - p) - np.log(a))


def get_ew(w0, w1):
    """Calculate the e(w) metric"""
    return (w1 - w0) / w0


def main():
    x = np.array([0, 0, 1, 1, 0, 1, 1, 1, 1, 0, 1, 1, 1,
                  0, 1, 1, 1, 0, 1, 1, 1, 1, 1, 1, 1, 1,
                  1, 1, 1, 1, 0, 1, 1, 1, 1, 1, 1, 1, 1, 1])
    p_baseline = 0.55
    p_enhanced = np.array([0.5, 0.5, 0.5, 0.5, 0.5, 0.5, 0.5, 0.5,
                           0.5, 0.5, 0.5, 0.5, 0.5, 0.5, 0.5, 0.5,
                           0.5, 0.5, 0.5, 0.5, 0.9, 0.9, 0.9, 0.9,
                           0.9, 0.9, 0.9, 0.9, 0.9, 0.9, 0.9, 0.9,
                           0.9, 0.9, 0.9, 0.9, 0.9, 0.9, 0.9, 0.9])
    a0 = ll(x, p_baseline)
    a1 = ll(x, p_enhanced)
    w0_p = get_w(a0)
    w1_p = get_w(a1)
    ew_p = get_ew(w0_p, w1_p)
    print('E(W) using scipy.optimize is: ', ew_p)


if __name__ == '__main__':
    main()
```

A Toy Example in MATLAB

```
x = [0, 0, 1, 1, 0, 1, 1, 1, 1, 0, 1, 1, 1, 0, 1, ...
     1, 1, 0, 1, 1, 1, 1, 1, 1, 1, 1, 1, 1, 1, 1, ...
     0, 1, 1, 1, 1, 1, 1, 1, 1, 1]; % truth

p_baseline = [0.55, 0.55, 0.55, 0.55, 0.55, 0.55, 0.55, 0.55, ...
              0.55, 0.55, 0.55, 0.55, 0.55, 0.55, 0.55, 0.55, ...
              0.55, 0.55, 0.55, 0.55, 0.55, 0.55, 0.55, 0.55, ...
              0.55, 0.55, 0.55, 0.55, 0.55, 0.55, 0.55, 0.55, ...
              0.55, 0.55, 0.55, 0.55, 0.55, 0.55, 0.55, 0.55];

p_enhanced = [0.5, 0.5, 0.5, 0.5, 0.5, 0.5, 0.5, 0.5, ...
              0.5, 0.5, 0.5, 0.5, 0.5, 0.5, 0.5, 0.5, ...
              0.5, 0.5, 0.5, 0.5, 0.9, 0.9, 0.9, 0.9, ...
              0.9, 0.9, 0.9, 0.9, 0.9, 0.9, 0.9, 0.9, ...
              0.9, 0.9, 0.9, 0.9, 0.9, 0.9, 0.9, 0.9];

% Calculate the log-likelihood
a0 = exp(sum(log(p_baseline) .* x + ...
    log(1 - p_baseline) .*(1 - x)) / length(x));
a1 = exp(sum(log(p_enhanced) .* x + ...
    log(1 - p_enhanced) .* (1 - x)) / length(x));

% Calculate 'w' using optimization
options = optimset('fmincon');
options.TolFun = 0;
options.TolCon = 1e-9;

% Function to be minimized
minimize_me = @(p, a) abs(p * log(p) + (1 - p) * log(1 - p) - log(a));

% Calculate 'w' using optimization
res0 = fmincon(@(p) minimize_me(p, a0), 0.5, [], ...
               [], [], [], 0.001, 0.999, [], options);
res1 = fmincon(@(p) minimize_me(p, a1), 0.5, [], [], ...
               [], [], 0.001, 0.999, [], options);

w0 = res0(1);
w1 = res1(1);

% Calculate the e(w) metric
ew = (w1 - w0) / w0;
fprintf('E(W) using fmincon is: %f\n', ew);
```

II.2 A fully worked example

Using data from the Titanic disaster (discussed below in S1-V.1), we calculate the IMV using two competing models predicting survival in the Titanic disaster. Specifically, we estimate two models:

$$\Pr(\text{death}_i) = \sigma(\beta_0) \tag{S3}$$

$$\Pr(\text{death}_i) = \sigma(\beta_0 + \beta_1 \text{sex}_i + \beta_2 \text{ticket class}_i) \tag{S4}$$

where σ is the logistic sigmoid. We replicate the process of a short, but standard analytical pipeline. In particular, we shuffle the dataset (with a seed set for reproducibility), and then compute the IMV associated with the increase in prediction quality associated with the second 'enhanced' model relative to the first. We do this across ten folds. Across these folds, the mean is 0.352 (SD of 0.143). Our accompanying code libraries also replicates this pedagogical example in MATLAB and Python for the benefit of users in those languages (see `https://github.com/crahal/InterModel_Vigorish`).

Predicting Survival on the Titanic with R

```r
library(readr)
set.seed(22092023)

ll<-function(x,p) {
  z<-log(p)*x+log(1-p)*(1-x)
  z<-sum(z)/length(z)
  exp(z)}

get_w<-function(a) {
  f<-function(p,a) abs(p*log(p)+(1-p)*log(1-p)-log(a))
  nlminb(.5,f,lower=0.001,upper=.999,a=a)$par}

minimize_me <- function(p, a) {
  return(abs((p * log(p)) + ((1 - p) * log(1 - p)) - log(a)))}

calculate_imv <- function(y_basic, y_enhanced, y) {
  ll_basic <- ll(y, y_basic)
  ll_enhanced <- ll(y, y_enhanced)
  w0 <- get_w(ll_basic)
  w1 <- get_w(ll_enhanced)
  return((w1 - w0) / w0)}

# Load and preprocess data
titanic <- read.csv('../data/titanic/titanic3.csv')
titanic$constant <- 1
titanic$sex <- ifelse(titanic$sex == "female", 1, 0)
titanic = titanic[sample(1:nrow(titanic)), ]
prev = mean(titanic$survived)
imv_list <- c()
k_folds <- 10

for (i in 1:k_folds) {
  # Calculate fold boundaries
  fold_size <- floor(nrow(titanic) / k_folds)
  fold_start <- (i - 1) * fold_size + 1
  fold_end <- round(i * fold_size)

  # Create train and test sets based on fold boundaries
  train <- titanic[-(fold_start:fold_end), ]
  test <- titanic[fold_start:fold_end, ]

  y_train <- train$survived
  y_test <- test$survived

  # Logistic regressions and predictions
  logreg_basic <- glm(survived ~ constant - 1,
                      data = train, family = binomial(link = "logit"))
  logreg_enhanced <- glm(survived ~ constant + pclass + sex - 1,
                         data = train, family = binomial(link = "logit"))

  pred_basic <- predict(logreg_basic,
                        newdata = data.frame(constant = test$constant),
                        type = "response")

  pred_enh <- predict(logreg_enhanced, newdata = test,
                      type = "response")

  imv_list <- c(imv_list, calculate_imv(pred_basic, pred_enh, y_test))
}

cat("IMV min:", min(imv_list), ", max:", max(imv_list),
    ", mean:", mean(imv_list), ", stdev:", sd(imv_list), ", prev: ", prev)
```

III Additional Simulation studies

III.1 The IMV versus Alternative Metrics

So as to compare the performance of the IMV, we introduce three alternative metrics. Using $y_i \in \{0, 1\}$ to denote the i-th observation of some binary outcome and $\widehat{p_i} \in (0, 1)$ as a prediction for that observation, we consider:

1. Pseudo-R^2 (subsequently, R^2): Motivated by recent work [7], we consider a metric inspired by the traditional R^2 metric of regression:

$$R^2 = 1 - \frac{\sum_i (y_i - \widehat{p_i})^2}{\sum_i (y_i - \bar{y})^2}. \tag{S5}$$

   This metric is closely related to the Brier score [8] which typically focuses on $\frac{1}{N}\sum_i (y_i - \widehat{p_i})^2$ where $i \in \{1, ..., N\}$.

2. AUC: The AUC (area under curve, [9]) metric has long been used to study performance of classifiers based on a comparison of the true and false positive rates.

3. F_1: The F_1 score [10] is the harmonic mean of recall (percentage of true cases that are correctly identified) and precision.

This is, of course, only a subset of the large number of potential metrics we could consider; we view these as being fairly popular and representative of a larger set.[2]

We begin by contrasting these fit statistics using the framework from [11] wherein we generate probabilities from two Beta distributions. Specifically, we sample four numbers from Unif$(1, 15)$. Denoting these as a_k, we generate 5000 probabilities from Beta(a_1, a_2) and 5000 probabilities from Beta(a_3, a_4). We then generate 10,000 based on these concatenated probabilities and we compute metrics using the known probabilities. Note that a large difference in the means of the two Beta distributions ($\frac{a_1}{a_1+a_2} - \frac{a_3}{a_3+a_4}$) should lead to better prediction by the model-based probabilities as compared to the overall mean. In Figure S2 we show these metrics for 1000 draws of a_k parameters alongside information about the difference between the expectations of the Beta distributions (where we've ordered things such that this difference is always positive) and the prevalence (based on $|0.5 - \hat{y}|$).

Note first that, as expected, all of the metrics are positively correlated with the difference in the means of the Beta distributions (i.e., $\frac{a_1}{a_1+a_2} - \frac{a_3}{a_3+a_4}$), thus indicating that they are sensitive to changes in the differences in probabilities for responses. The indices are sensitive to prevalence in the sense that there is more variation in the metrics when $|0.5 - \hat{y}|$ is nearer to 0. The metrics are all positively correlated amongst themselves. In particular, the AUC and R^2 statistics closely mirror each other. The lowest correlation between the IMV and another metric is with the F_1 score ($r = 0.35$). This is driven by the fact that the F_1 score shows peculiar behavior as a function of prevalence (note the "spike" in that scatterplot); a potential problem with usage of this statistic.

III.2 Fit statistics as a function of prevalence

Below we make use of the following equation to simulate data:

$$\Pr(y_i = 1) = \sigma(\beta_0 + \beta_1 x_i + \beta_2 z_i) \tag{S6}$$

where $\sigma(\cdot)$ is the logistic sigmoid ($\sigma(x) = (1 + \exp(-x))^{-1}$) and x_i, z_i are independent samples from Normal$(0, 1)$. For each iteration of the simulation, we first generate triplets $(\beta_0, \beta_1, \beta_2)$. The predictors x and z are then independently drawn from the standard normal distribution, with y being drawn from a Bernoulli distribution with probability specified by Equation S6. This process is repeated to generate observations (x_i, z_i, y_i) for $i \in \{1, ..., 4000\}$.

We begin with an examination of the fit statistics under different assumptions about the prevalence of the underlying binary indicator. We consider results based on 1000 simulations wherein β_0, β_1 are independently

[2] We separately consider the AIC below given that it produces values on a different scale.

sampled from Unif[0, 1] and $\beta_2 = 0.3$. Here, we blind ourselves to z and predict based on x alone. For IMV, the comparison is to prediction based on prevalence alone. Results in the form of a LOWESS line (with 95% confidence intervals) to the 1000 points, showing patterning as a function of β_0 and β_1 are shown in Figure S3. As expected, all metrics are increasing as a function of β_1. However, note that there is varying sensitivity to values of β_0. AUC and R^2, for example, are unassociated with β_0 (echoing what we observed in Section 3 of the main text). Whether this is desirable is a crucial question; from our perspective, the IMV provides a clear answer. Larger values of β_0 are consistent with less stochastic outcomes and consequently there is less to be gained (in terms of our single blind bet) from knowledge of x. This is apparent in the decrease of IMV as a function of β_0.

III.3 Variation in the IMV across folds

We conduct a small simulation study to ensure that the SD of the ω values computed across the $k = 10$ folds is a reasonable method for analyzing sampling-related uncertainty in the IMVs computed via the mean ω across the folds. We simulate outcomes based on $\Pr(y = 1) = \sigma(\beta x)$ for $\beta \in \{0.1, 0.5\}$, $x \sim \text{Normal}(0, 1)$, and 250 choices of sample size. In each of the k folds we compute the IMV—denoted ω_k to emphasize that it is fold-specific—based on training of a model in the other folds and prediction in the focal fold.

Results are shown in Figure S4. On the left, we show the resulting parametric standard error based on estimating a logistic regression model. At right, we compute the SD of the ω_k values computed over the $k = 10$ folds. These SDs decline in a manner similar to that of the parametric standard errors thus suggesting that the variation in the ω_k values is a reasonable way of indexing sampling-related uncertainty in our overall estimates of the IMV (i.e., $\overline{\omega_k}$).

III.4 The IMV and overfitting

To emphasize the importance of the train and test split, we use a simulation study to show that the IMV can identify overfitting in test data. This example builds on the observation in Figure 5 showing that the IMV can be used to detect overfitting but here we additionally show that while the IMV prefers more complex models in training data (as expected given that the IMV is based on the unpenalized log-likelihood) but does not have this property when computed with test data.

In this simple example, we simulate data based on $\Pr(y_i|x_i) = \sigma(0.5x_i)$ where $x_i \sim \text{Normal}(0, 1)$. We then fit a model that is linear in x,

$$\Pr(y_i|x_i) = \sigma(b_0 + b_1 x_i) \tag{S7}$$

and a model that includes both linear and quadratic terms in x

$$\Pr(y_i|x_i) = \sigma(c_0 + c_1 x_i + c_2 x_i^2). \tag{S8}$$

For a given sample size, we simulate values of x_i and then produce training and test versions of y_i via the true model. We randomly generate 10000 sample sizes and compute the IMV for predictions in test data based on the linear and quadratic fits. Results are shown in Figure S5. When we compute the IMV using training data (i.e., the data used for model fitting), the quadratic fit is uniformly preferred (although gains are quite small for larger samples). However, when we use test data (as done in the main body of the paper), the IMV is negative suggesting that the predictions from the quadratic model are worse than those from the linear model (as expected given that the latter is in fact the true model).

Figure S2: Comparison of four metrics, along with the difference ($\frac{a_1}{a_1+a_2} - \frac{a_3}{a_3+a_4}$) and prevalence ($|0.5 - \hat{y}|$), based on data simulated from Beta distributions.

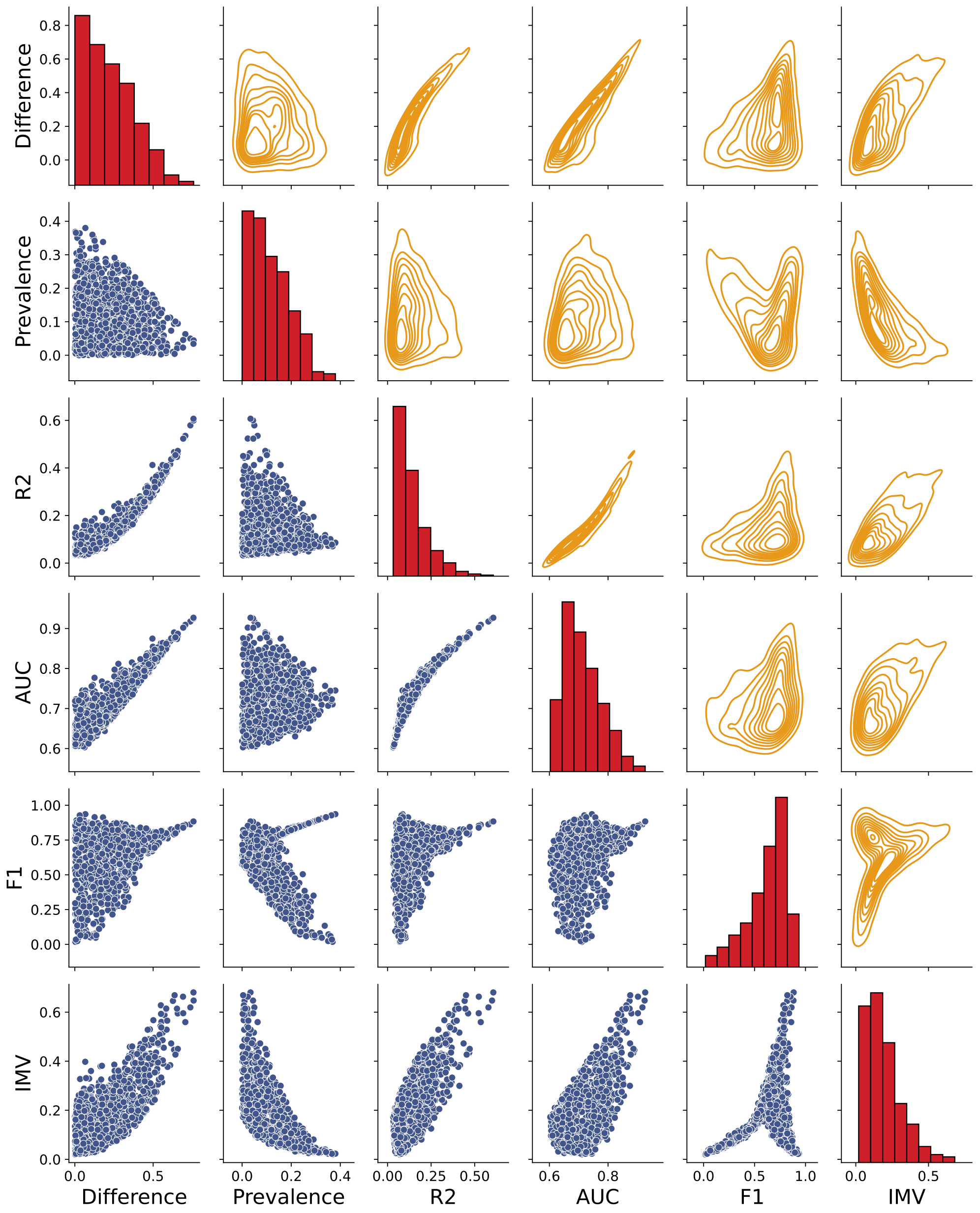

Figure S3: Patterning of fit metrics based on x alone. Metrics are fit as a function of β_0 and β_1 ($\beta_2 = 0.3$). Curves are fitted with a LOWESS algorithm, and dashed lines represent 95% Confidence Intervals.

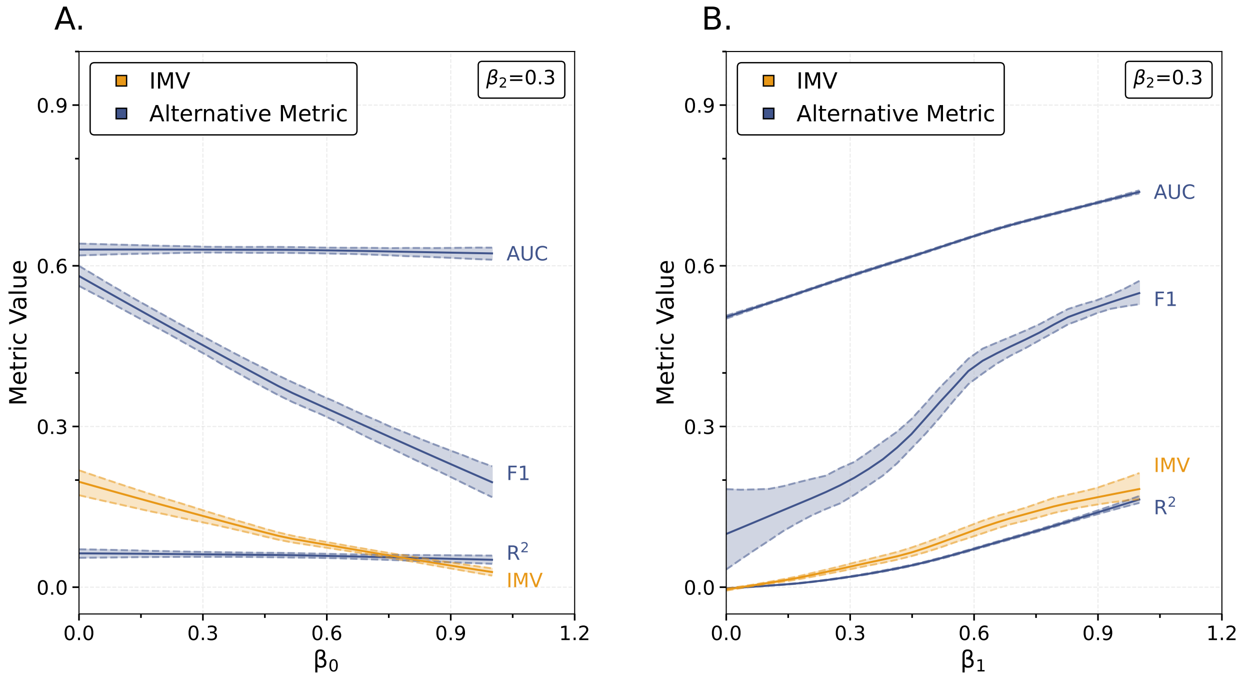

Figure S4: Comparison of standard error of parameter estimate in logistic regression model (left) versus variation in ω values across $k = 10$ folds for different choices of sample size.

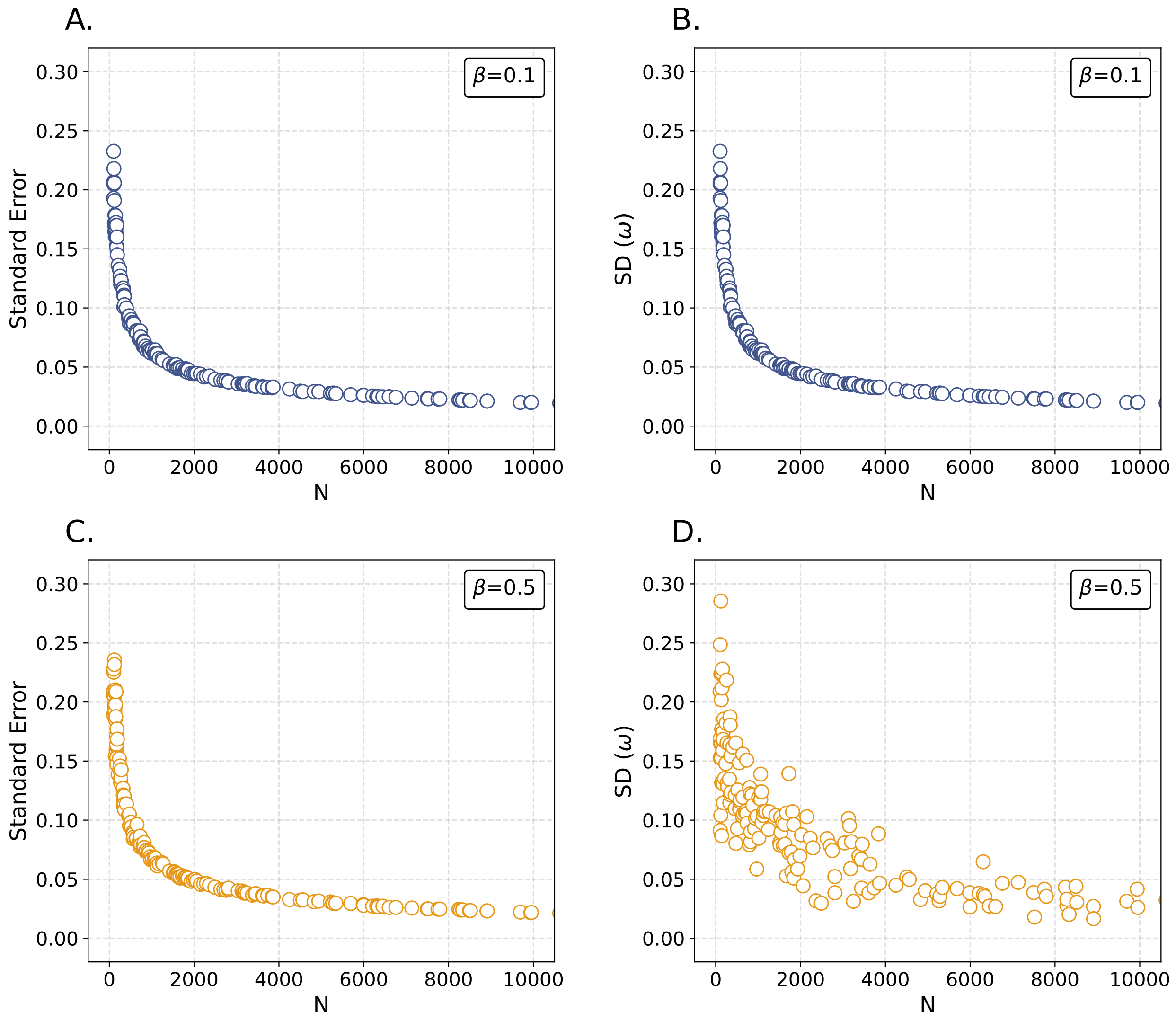

Figure S5: Comparison of linear versus quadratic models applied to data generated from linear model when computed in training versus test data. We vary sample size and generate outcomes via $\Pr(y = 1|x) = \sigma(0.5x)$ where $\sigma(\cdot)$ is the standard logistic sigmoid and $x \sim \text{Normal}(0, 1)$.

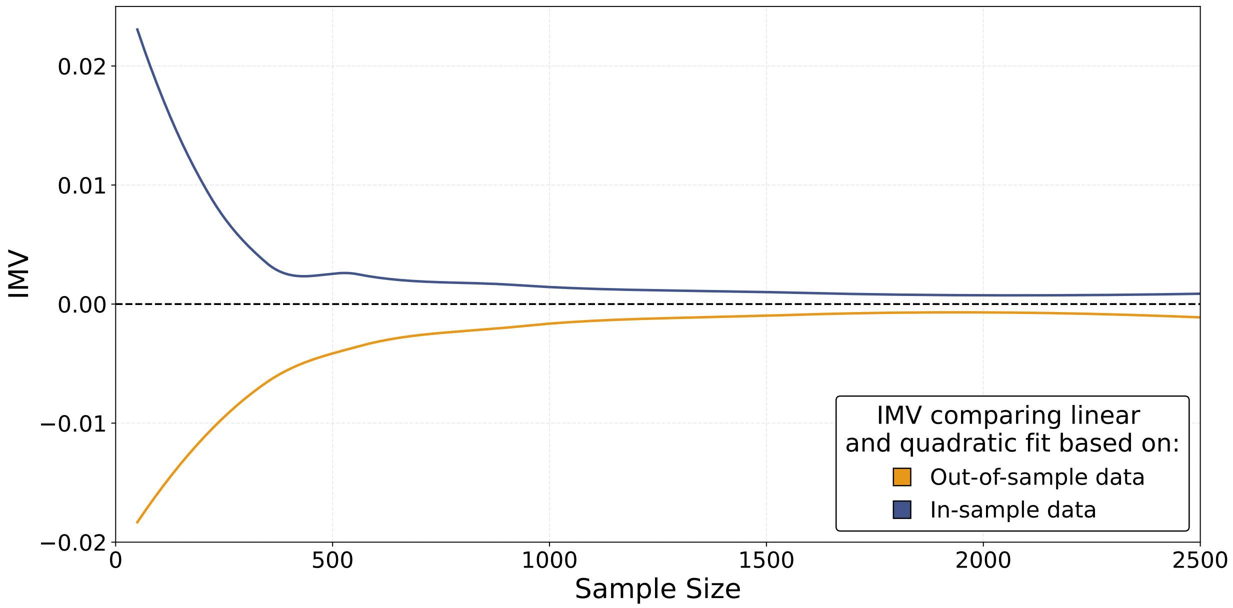

IV Additional Details on Core Empirical Illustrations

IV.1 The Health and Retirement Study

The US-based Health and Retirement Study (HRS, [12, 13]) is a biennial longitudinal study of US adults over 50. The HRS collects data on both social status as well as health. We use data from the RAND Corporation's 2016 HRS data release.[3] We focus on observations on those aged 60 and over. Across all waves, there are 100,243 such observations. Sample sizes varied by age and outcome. We only compute data in cases with at least 1000 respondents. Sample sizes ranged from 1188 to 15864 (mean of 7582). We use logistic regression to predict outcomes based on linear combinations of the indicated predictors. We focus on the prediction of health outcomes *at the next wave* within an age bin (those within 1.5 years of a focal age) based on predictors measured at the current wave (or that are time-invariant).

We focus on prediction of self-reported physical health measures. At each wave, respondents are asked if they have been diagnosed with any of the following: high blood pressure, diabetes, cancer, lung disease, heart problems, stroke, psychological problems, arthritis. We also ask whether the respondent died prior to the next wave or had information completed by a proxy respondent (which typically occurred due to substantial impairment of the respondent's physical or mental functioning). To predict these outcomes, we rely on demographic information (age, sex, race/ethnicity), years of education reported by the respondent (a time-invariant predictor given the age of the respondent), and two specialized measures that the HRS collects.

- Cognition: The HRS measure of cognitive functioning [14] is based on the TICS survey phone-based assessment of cognitive status [15]. We used the `cogtot` measure constructed by RAND [15] for respondents who did not respond via proxy. The measures summarize word recall (via immediate and delayed recall of a 10 word list) and mental status (serial 7s, backward counting, correct naming of objects, memory, and date).
- Grip strength & Gait speed: The HRS collects measures of grip strength and gait speed (details on measurement can be found in [16]). These are only available at every other wave for each respondent given the structure of the HRS interview (which alternates between in-person and phone-based data collection). Gait speed was only collected in those 65 and older.

Prevalences as a function of age are shown in Figure S6. The outcomes, shown in Panel A, generally increase as a function of age although such increases may be relatively slight. The predictors, Panel B, show more dramatic changes. Gait speed decreases dramatically across age (resulting in increased time) while grip strength and the score on the cognitive test both decline across age. We first predict health outcomes based on demographics (sex and race) relative to prevalence alone (Panel A), then add educational attainment as a predictor (Panel B). In Panels C and D, we turn to predictions based on relatively expensive-to-collect pieces of health data: cognition and physical functioning (as proxied for by grip and gait).

IV.2 The General Social Survey

The General Social Survey (GSS, [17]) is a survey of the social attitudes of Americans that has run since 1972.[4] In particular, it is widely used to understand the changing nature of political ideologies in the US. In total, there are 64,816 observations. Sample sizes varied across years; the smallest sample was 1193 in 1990 and the largest was 3385 in 2006. We focus on prediction of political party affiliation for partisans (the US has two major political parties). We consider independents who report leaning in one direction as partisans in that direction at that point in time (but exclude those who report no lean). Predictions are based on logistic regression models based on respondent demographics: age, sex, and whether the respondent identifies as white (as opposed to black or other) versus prevalence alone. The proportion of the sample classified as Republican is shown in the associated figure of the main text (bottom panel); note that comparisons of predictive accuracy over time will need to account for variation in this proportion.

[3] The data is publicly available, `https://www.rand.org/well-being/social-and-behavioral-policy/centers/aging/dataprod.html`; note that the grip and gait data come from the public release HRS files.

[4] The GSS data is publicly available, `https://gss.norc.org/get-the-data`.

Figure S6: Age trends in HRS measures

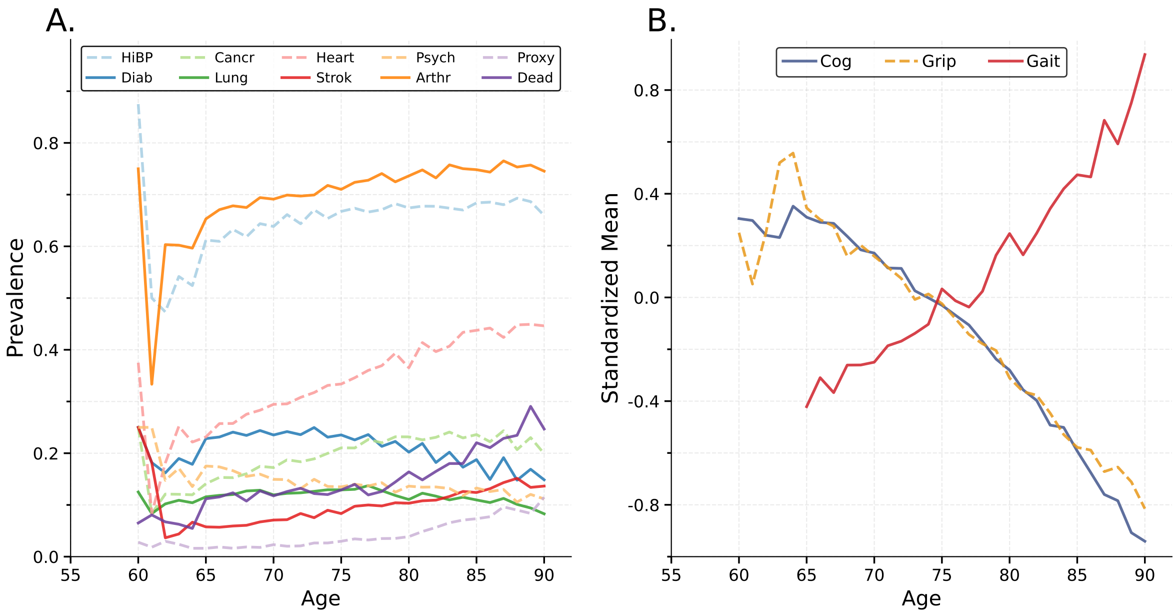

IV.3 The Fragile Families Challenge

We utilize the replication materials—which include individual team submissions—from the FFC itself [18] in conjunction with the freely available FFC data.[5] Here we focus on the three binary outcomes. We re-estimate their baseline models for binary outcomes (with logistic regressions as opposed to linear probability models, see [7]), drop contributed submissions which took the form of class labels, and keep only those submissions which have a $R^2 > 0$ (i.e. outperform the prediction made by a simple training mean).[6] We then calculate the IMV metric for the same test-split that was used in the original challenge itself, which represents a single fold only. Note: minor variation exists with the results reported in the FFC paper unless the Docker image is used.

[5]We specifically use the `FFChallenge_v5.zip` data, available from Princeton's Office of Population Research. Replicating our utilization of the IMV within the FFC replication materials requires a simple and accessible registration at `https://pop.princeton.edu/`.

[6]This results in 95 submissions for 'layoff', 92 for 'jobTraining', and 88 for 'eviction'.

Figure S7: IMV for predicting health at next wave as a function of different sets of predictors. Panel A shows prediction based on sex and race relative to prevalence alone while Panel B shows prediction based on years of education added to race and sex. Panel C shows prediction based on cognition relative to race, sex, and education and Panel D the prediction based on grip and gait added to race, sex, and education.

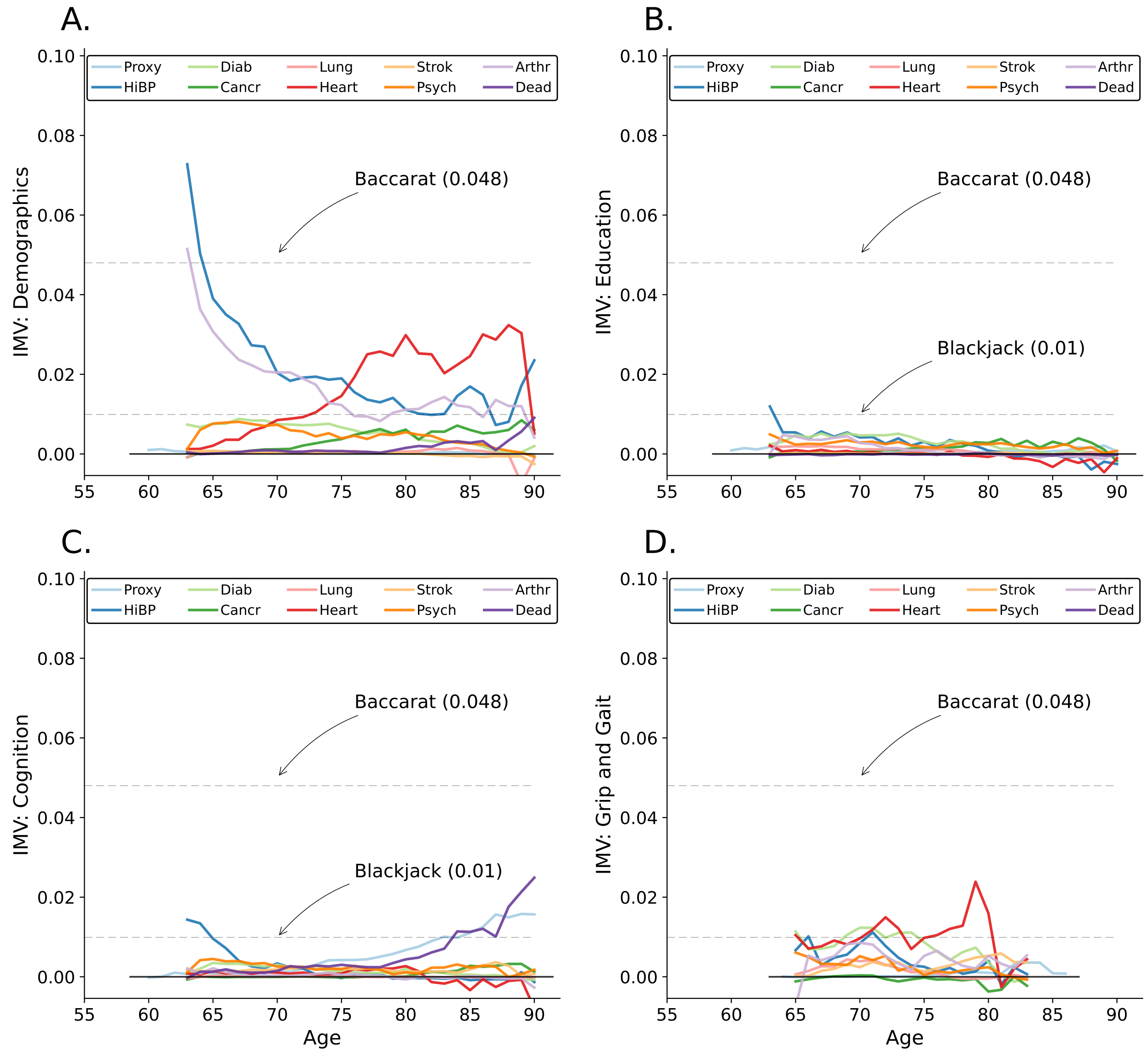

V Additional Empirical Examples

V.1 Survival on the Titanic

V.1.1 Logistic Regression Estimates

We use predictions of death in the Titanic disaster to offer an example of how the IMV can be used to generate additional insight into coefficients from logistic regression. Logistic regression coefficients are challenging to directly interpret or compare [19, 20]. When interest is in the coefficient for a given covariate, our suggested approach is to fit logistic regression models with and without that covariate. These models play the role of the enhanced and baseline model; the resulting IMV pertains to the predictive value of the covariate.To illustrate this point, we focus on sex as a predictor of death amongst Titanic passengers. As a contrast, we consider the role of sex in predicting political affiliation, specifically a tilt towards the GOP in the GSS [17]. The GSS sample is far larger and split more evenly by sex (see summary in Table S1). Note that the outcomes occur at roughly similar rates (62% for the Titanic and 59% for the GSS).

Logistic regression estimates (β, see Table S1) of association (net of other controls) between being female and the outcome are -2.64 (SE=0.22) in the Titanic data and 0.21 (SE=0.02) in the GSS. How to directly compare these coefficients in terms of the key question—how much does information about sex resolve uncertainty?—is non-obvious. Our baseline model (excluding sex) is much more predictive of outcomes in the Titanic data; differences in the predictive value of sex aren't due to the fact that the baseline model for Titanic deaths is qualitatively weaker than the baseline model in the GSS. Based on the IMV, sex in the Titanic case is far more valuable, $\omega = 0.136$, than sex in the GSS, $\omega = 0.00239$. In our view, this quantity offers an unambiguous interpretation: in terms of literal value, having access to sex in the prediction of individual outcome (i.e., death) in the Titanic data is 57 times as valuable as this same data on the GSS respondents for prediction of those outcomes (i.e., political affiliation).

Table S1: Illustration of IMV as a means of understanding logistic regression coefficients.

	N	Proportion Female	Outcome Prevalence	β	ω_0 [a]	ω
Titanic	891	0.35	0.62	-2.64	0.29	1.36e-1
GSS	64816	0.56	0.59	0.21	0.14	2.39e-3

[a] ω_0 is the IMV for the model without sex compared to prediction based on outcome prevalence alone.

As a contrast to the results of Table S1, we also include some conventional metrics of model fit for the various models considered here in Table S2. These metrics indicate that information about sex of the respondent leads to improvements in prediction; moreover, improvements seem more pronounced in the Titanic example (although the dependence of AIC values on sample size make a quick interpretation of this fact difficult). However, the problem of portability is apparent as the differences in the various metrics across models lack clear interpretation.

Table S2: Alternative metrics for comparing two models (i.e., prediction of outcomes with/without sex as in Table S1).

		R^2	AUC	AIC
Titanic	No sex	0.21	0.76	825.32
	w/ sex	0.42	0.86	650.68
GSS	No sex	0.08	0.63	66911.33
	w/ sex	0.08	0.64	66788.38

Table S3: Illustration of the IMV relative to other metrics in a Kaggle-style competition.

	Predictive Approach					
Metric	Logistic	SVC	Naive Bayes	LightGBM	RF	Prevalence
Log Loss	0.4489	0.4490	*0.6248*	**0.4342**	0.4968	0.6666
Accuracy	0.8069	0.8092	*0.7676*	**0.8372**	0.8283	0.6162
Brier Score Loss	0.1419	0.1408	*0.1786*	**0.1319**	0.1370	0.2368
F_1	0.7407	0.7351	*0.7208*	**0.7716**	0.7602	0.0000
ROC-AUC	0.8599	0.8493	*0.8429*	**0.8649**	0.8593	0.5000
Jaccard	0.5903	0.5843	*0.5660*	**0.6303**	0.6161	0.0000
R^2	0.3999	0.4053	*0.2435*	**0.4416**	0.4194	0.0000
IMV	0.0000	-0.0004	*-0.1815*	**0.0099**	-0.0516	-0.2763

Bold entries indicate the best, and italicized indicates the worst performing models as assessed by each metric. The 'Prevalence' predictions are simply a vector of the mean of the dependent variable in the training set (i.e. $\hat{y}_i = \bar{y}_{\text{train}} \forall i$) for reference. For the Log Loss and Brier Score Loss metrics, a lower score indicates superior performance. IMVs are calculated with the predictions generated by the Logistic model as the 'baseline'.

V.1.2 Prediction Competitions

We use publicly-available information on the passengers (e.g., gender, ticket type, etc.) aboard the Titanic when it sunk.[7] While the majority of the 891 passengers were male (65%), an even larger share of the 549 passengers who died were male (85%). For the prediction of death in the Titanic data, we use information on the ticket class, age, number of siblings/spouses onboard, number of parents/children onboard, and the port of embarkation alongside sex. In the parallel prediction of party membership in the GSS, we add age, marital status and race to sex.

We also use the Titanic dataset to show the that the IMV can be utilized as a more interperable ranking metric in something akin to Kaggle competitions, where the choice of evaluation metric has been a point of significant contention historically. We argue that by using the IMV across different predictive problems, we are not only able to generate a significantly cleaner interpretation of just how predictive certain tasks are, but we are able to make more informative and direct comparisons of different predictive approaches (whether approaches differ in their usage of features or their underlying algorithm) to a common task. We undertake some light additional feature engineering (such as creating age bins, cleaning honorifics, and so forth) and then apply four commonly utilized machine learning tools (a Support Vector Classifier, Guassian Naive Bayes, Light Gradient Boost, and the Random Forest). We compute a variety of potential evaluation metrics—including both probabilistic and class based metrics—after building models using stratified 10-fold cross validation with approximately equal folds. We take the mean across the folds for each metric. For the IMV, we focus on the value computed from predictions from the algorithm in question versus predictions generated by the Logistic model.

Results are shown in Table S3, where in addition to the IMV, we also calculate the R^2, ROC-AUC, F_1, Accuracy, the Log Loss (which is simply $-(y_i \log(\widehat{y_i}) + (1-y_i)\log(1-\widehat{y_i})))$, and the Brier Score [8]. Across all metrics, the LightGBM is consistently ranked as the best predictor while the Gaussian Naive Bayes ranks as the worst. There are small variations in rankings across the metrics. The IMV, for example, suggests that the Random Forest approach offers less predictive value than either logistic regression or the SVC (as does the log loss). In contrast, the Accuracy, F_1, and the AUC-ROC all rank the random forest as superior to those two. While the LightGBM model performs the best in terms of accuracy (the common metric of choice for this competition), the IMV offers useful intuition about the degree to which it outperforms logistic regerssion. Here, predictive improvements due to this particular modeling strategy are relatively modest ($\omega = 0.0099$). Model-based improvements in predictions may be far larger in other cases (i.e., other prediction competitions) and the portability of the IMV allows for such a comparison.

[7]Data from `https://www.kaggle.com/c/titanic`.

Figure S8: An IMV based re-analysis of the 'COVID Symptom Study' [21]. Panel A shows the IMV plotted against the ROC-AUC across each of 100 folds estimated with the 'small' model based on the First Year. Panel B shows the IMV across folds of predictions for the small and large model in both the First Wave and First Year of data.

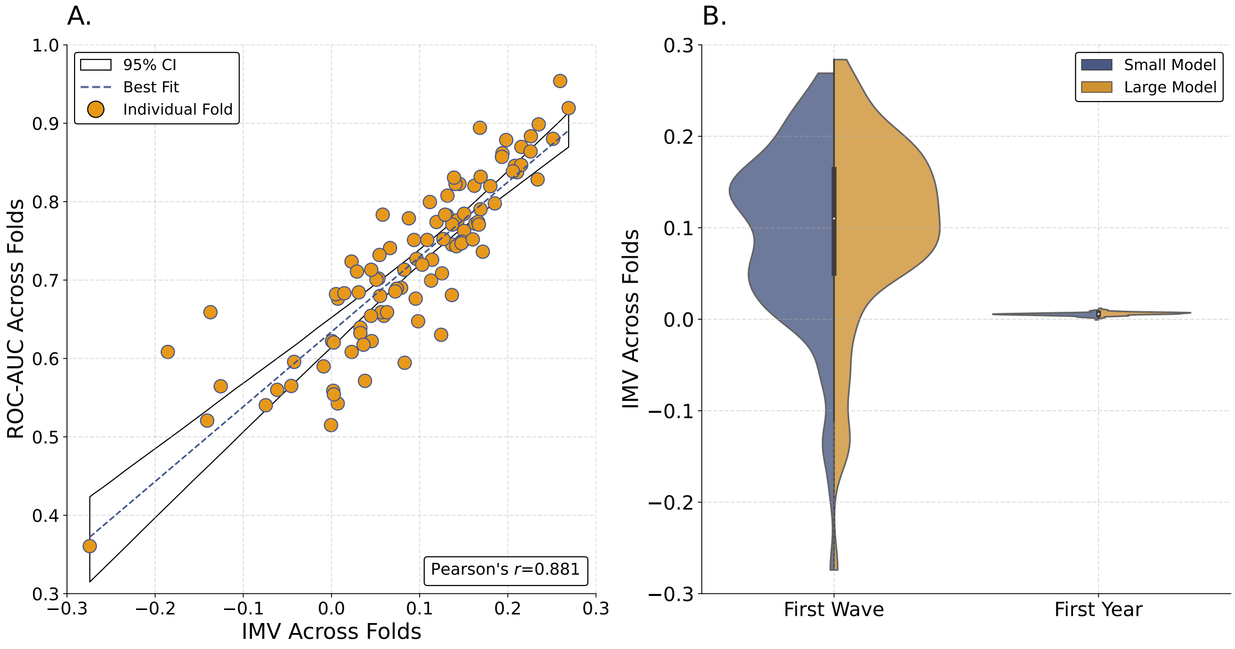

V.2 The COVID-19 Symptom Study

We attempt to predict positive COVID-19 cases utilizing data from the 'COVID Symptom Study' [21], a smartphone-based app previously known as the 'COVID Symptom Tracker'. Designed to collect anonymous data and track self-reported symptoms of COVID-19 in real-time, the app played an essential role in initially establishing the loss of taste and smell as a primary symptom of infection. We use data for both the first month of the app's availability (24th March 2020 through 23rd April, 2020; the 'First Wave') and almost a full year through to the 24th of March, 2021; note that the full first year was not analyzed in the original study [21]. These two time periods represent different paradigms of test availability, sample size (n=4527 and n=427588 for the first month and the first year respectively), and prevalence of positive results (0.31 and 0.06). We focus on a logistic regression model that is consistent with the focal model in [21] and consists of age, sex, and some key symptoms.

We emphasize three points via Figure S8. First, in each fold, the IMV is strongly correlated with the ROC-AUC. This is consistent with our simulation evidence that suggests these metrics provide similar information in some scenarios (though the AUC and IMV do differ in key ways; consider for example the differential sensitivity to prevalence noted elsewhere). Second, note a change in the degree to which the reported symptoms predict COVID. In the first month of COVID, symptoms were strongly predictive (mean IMV of 0.092 for our focal 'small' model). During this time, limited tests led to availability only to those exhibiting symptoms. However, as tests became widely available in the first full year and behavior related to usage of tests changed, the same set of predictors became far less valuable as a predictor of a positive test. This change in the IMV suggests an important shift in the way that the sentinel symptoms of COVID are predicting positive diagnosis during different paradigms of test availability. Third, the incorporation of additional information in the 'large' model provided no substantive increase in predictive accuracy during either period.

COVID Data were collected via a smartphone application; these data are available to researchers within and outside of both academia and the National Health Service (NHS) through the NHS-funded Health Data Research UK (HDRUK)/SAIL consortium. One of the primary applications of the dataset has been the

creation of clinical prediction tools, as done elsewhere via an unsupervised time series clustering algorithm [22]. We extract the necessary data from three tables of the COVID-19 Symptom Study SQL database: Patients, Assessments (symptoms), and Tests. We then filter variables in the same way as the original work (age: 16-90 years; height: 110–220cm; weight: 40–200kg; BMI: 14–45kg/m^2; and temperature: 35–42°C). We ensure that a minimum of nine of the ten symptoms questions are answered, and that the question pertaining to the loss of taste and smell specifically is answered, in line with the original study. We also undertake a cursory check which ensures that all patients are featured in each three of the database views. If multiple tests are taken on the same day, we collapse to any positive result, or negative otherwise. We then generate windows based on the dates of tests. If the previous test was over 14 days ago, we take the most recent 14 days as the window for symptom reporting. If the previous test was taken under 14 days ago, we set that period as the window. If we observe any positive report of a symptom for any assessment undertaken in that window (where windows relate to individual tests, unless there are multiple on one day), then that symptom is coded as positive.

As in the original paper, we consider predictions from logistic regressions using information on age, sex, asmosia, coughing, fatigue, skipping meals (our 'small' model). In a second 'large' model, we then include BMI, shortness of breath, fever, delirium, diarrhoea, hoarseness, chest pain, and abdominal pain. We are able to obtain estimates largely consistent with those of the original paper: with the 'small' model and the initial month of data, we estimate an Odds Ratio for loss of sense of taste and smell of 5.462 (6.74 in the original paper) and generate a 5-fold AUC-ROC of 0.719 (0.76 in the original paper). Results in the main text are taken over 100 stratified and approximately equally-sized folds.

V.3 OECD's Programme for International Student Assessment (PISA)

PISA is an international assessment conducted every three years and aims to measure "the extent to which 15-year-old students, near the end of the compulsory education, have acquired key knowledge and skills"[23]. We use data from the students who took the 2015 PISA math exam.[8] We use a random sample of 10,000 15 year olds from the 2018 math assessment. We estimate item response theory (IRT) models via the EM algorithm and then obtain EAP ability estimates (both from [24]; we focus on EAP ability estimates and place priors on discrimination and guessing parameters). Predictive fit is based on the "missing response" paradigm [25]; that is, we hold item responses as test data and attempt to predict them based on item- and person-level features derived from analysis of training data.

PISA uses the two parameter logistic model (2PL) based on previous considerations of fit [26]; we compare the fit of the 2PL to fit based on the 3PL and the 2 factor 2PL. We first consider gains from more flexible models relative to the 2PL. For the 3PL, the IMV based on 10-fold CV was 0.0019. For the two factor 2PL, $\omega = -0.0006$ indicating that the two factor 2PL does a worse job with prediction of test data than does the 2PL. Gains from these more flexible approaches as compared to the currently used 2PL are thus quite small. As one way of contextualizing this, going from the previously used 1PL model to the 2PL generated $\omega = 0.01$; an order of magnitude larger than going from the 2PL to the 3PL.

V.4 Predicting family income from text data

To further illustrate the flexibility of our metric, we build off of recent work examining interplay between income and the kinds of themes identified by topic modeling in a large corpus of essays submitted as part of college applications [27]. In particular, we consider the IMV associated with prediction of whether a reported family income is above or below the median; in these data, the median family income across the 59723 respondents is $70,000. Given the longstanding observation regarding associations between income and the SAT [28], we inquire about the degree to which the 70 themes predict this indicator net of the applicant's total SAT score. In these data, the correlation between the SAT score and the indicator is 0.43. Across 10-fold CV, the IMV associated with the topics is 0.073.

[8] PISA data is publicly available, `https://www.oecd.org/pisa/data/`.

V.5 Predicting whether home team wins football in professional European competitions

We consider the year wherein the IMV is highest using data and predictions from [29]. Across all years and leagues, the average IMV was 0.16. The general increase in the model's power for predicting the winner is seen in the increase in the IMV over time; the highest IMV ($\omega = 0.244$) was observed in 2017 when 61% of home teams won their matches. As points of contrast, we also consider results for England and the Netherlands. The results for England show a similar pattern while, as in [29], the results for the Netherlands show less change over the period observed here.

V.6 Raw prediction examples

To offer additional benchmarks for interpretation of IMVs, we consider a range of raw predictions (meaning we are merely interested in the quality of prediction—which the IMV facilitates—rather than relative improvement in prediction) using a variety of different empirical datasets taken from [30]. Unless otherwise specified, we are computing the IMV based on 10-fold CV using logistic regressions (where covariates are included additively) as compared to prediction based on prevalence in the out-of-fold data. Additional documentation can be found in the manuscript's accompanying software library (see `https://github.com/crahal/InterModel_Vigorish`).

- Prediction of whether a given sample of glass was produced via a "float" process (useful for forensic identification of samples) in 214 samples [30]. Prediction is based on the sample's refractive index and results of chemical assays and produce $\omega = 0.420$ compared to prediction based on prevalence alone.
- Prediction of whether the number of rings in a sample of 4176 abalone is above or below the median [31]. Prediction is based on a variety of anthropometric characteristics (e.g., diameter, weight) and sex. The IMV was $\omega = 0.667$.
- Early prediction of diabetes using patient reported symptoms (e.g., itching, age, sex, obesity, etc.) from a survey of 520 respondents [32]. The IMV was $\omega = 0.617$.
- Diagnosis of breast tissue as malignant or benign based on a subset of imaging characteristics in 569 tissue samples [33]. The IMV was $\omega = 0.526$.
- Excess alcohol consumption (dichotmization as per the rule in [34]) based on 6 blood tests from 345 blood samples [35]. The IMV was $\omega = 0.245$.
- Identification of whether a pixel is a skin tone or not based on B,G,R values [36]; prediction based on both main effects and all possible interactions between these three predictors in 245057 pixels. The IMV was $\omega = 0.196$.
- Diagnosis of heart disease (based on angiographic disease status) in a clinical sample of 302 patients based on 14 predictors [37]. The IMV was $\omega = 0.123$.
- Hospital readmissions for patients with Diabetes Mellitus (DM) based on a subset of their medical record from a previous hospital visit (e.g., number lab procedures, number medications) in 101766 hospital visits [38]. The IMV was $\omega = 0.196$.
- Lithofacies class (out of 9 classes) amongst 3232 well samples using well-log data [39]. We focused on predictions of each class versus all others and each class versus adjacent classes (see Table 2 in [39]). When predicting classes versus all alternatives, the values ranged from $\omega = 0.163$ for predicting Nonmarine coarse siltstone and $\omega = 0.016$ for predicting Dolomite (which was the rarest of the classes, only occurring in 3% of the well samples). Predicting Dolomite versus the adjacent facies classes of Wackestone or Packstone-grainstone was similarly the weakest of these predictions with $\omega = 0.049$. Predicting Marine siltstone and shale versus the adjacent class of Mudstone was the largest IMV with $\omega = 0.446$.

Figure S9: Prediction of whether home team wins using model from [29]. Blue dots represent points from individual years while the golden lines represent LOWESS fits.

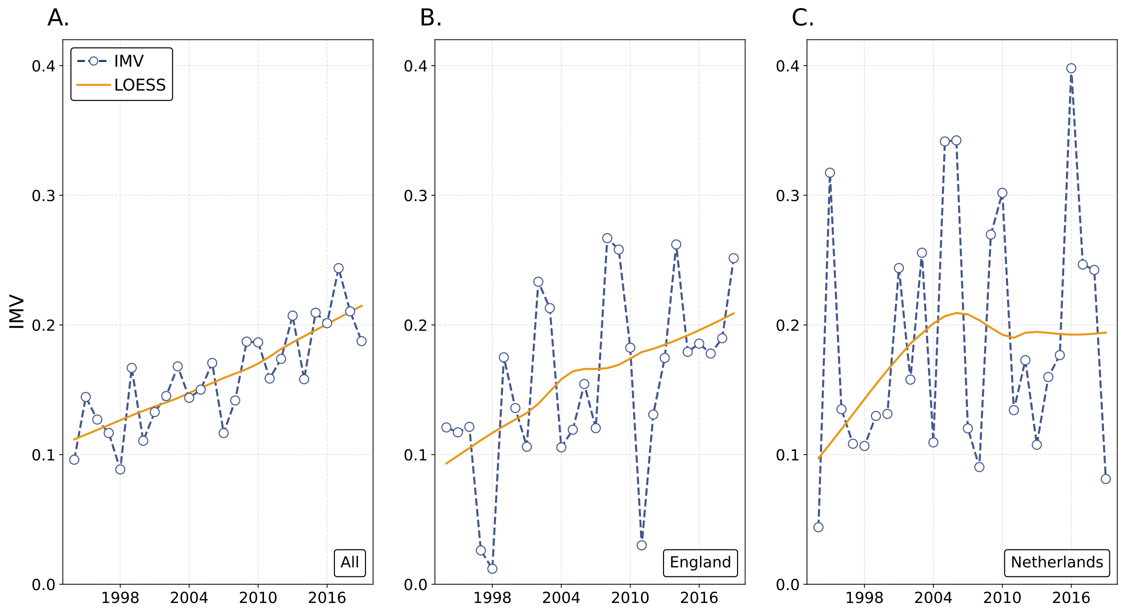

References

[1] Tilmann Gneiting and Adrian E Raftery. Strictly proper scoring rules, prediction, and estimation. *Journal of the American statistical Association*, 102(477):359–378, 2007.

[2] Ronald A Howard. Decision analysis: Perspectives on inference, decision, and experimentation. *Proceedings of the IEEE*, 58(5):632–643, 1970.

[3] Corey A Shank. Is the nfl betting market still inefficient? *Journal of Economics and Finance*, 42(4):818–827, 2018.

[4] Jon P Morosky. Method of administering and playing a baccarat type card game, May 23 2000. US Patent 6,065,753.

[5] Kurt Lofink and Richard Lofink. Blackjack game with modifiable vigorish, April 17 2001. US Patent 6,217,024.

[6] John L Kelly Jr. A new interpretation of information rate. *The Bell System Technical Journal*, 34(4), 1956.

[7] Matthew J Salganik, Ian Lundberg, Alexander T Kindel, Caitlin E Ahearn, Khaled Al-Ghoneim, Abdullah Almaatouq, Drew M Altschul, Jennie E Brand, Nicole Bohme Carnegie, Ryan James Compton, et al. "Measuring the predictability of life outcomes with a scientific mass collaboration": Correction. *Proceedings of the National Academy of Sciences of the United States of America*, 118(50), 2021.

[8] Glenn W Brier et al. Verification of forecasts expressed in terms of probability. *Monthly weather review*, 78(1):1–3, 1950.

[9] Jin Huang and C.X. Ling. Using auc and accuracy in evaluating learning algorithms. *IEEE Transactions on Knowledge and Data Engineering*, 17(3):299–310, 2005.

[10] Alex P Zijdenbos, Benoit M Dawant, Richard A Margolin, and Andrew C Palmer. Morphometric analysis of white matter lesions in mr images: method and validation. *IEEE transactions on medical imaging*, 13(4):716–724, 1994.

[11] Davide Chicco, Matthijs J Warrens, and Giuseppe Jurman. The matthews correlation coefficient (mcc) is more informative than cohen's kappa and brier score in binary classification assessment. *IEEE Access*, 2021.

[12] F Thomas Juster and Richard Suzman. An overview of the health and retirement study. *Journal of Human Resources*, pages S7–S56, 1995.

[13] Amanda Sonnega, Jessica D Faul, Mary Beth Ofstedal, Kenneth M Langa, John WR Phillips, and David R Weir. Cohort profile: the health and retirement study (hrs). *International journal of epidemiology*, 43(2):576–585, 2014.

[14] Mary Beth Ofstedal, Gwenith G Fisher, A Regula Herzog, et al. Documentation of cognitive functioning measures in the health and retirement study. *Ann Arbor, MI: University of Michigan*, 10, 2005.

[15] Delia Bugliari, Nancy Campbell, Chris Chan, Orla Hayden, Michael Hurd, Regan Main, Joshua Mallett, Colleen McCullough, Erik Meijer, Michael Moldoff, et al. Rand hrs data documentation, version p. *RAND Center for the Study of Aging*, 2016.

[16] Eileen Crimmins, Heidi Guyer, K Langa, Mary Beth Ofstedal, Robert Wallace, and D Weir. Documentation of physical measures, anthropometrics and blood pressure in the health and retirement study. *HRS Documentation Report DR-011*, 14(1-2):47–59, 2008.

[17] James A Davis and Tom W Smith. *The NORC general social survey: A user's guide*, volume 1. SAGE publications, 1991.

[18] Matthew Salganik, Ian Lundberg, Alex Kindel, and Sara McLanahan. Replication materials for "Measuring the predictability of life outcomes using a scientific mass collaboration", 2020.

[19] Richard Breen, Kristian Bernt Karlson, and Anders Holm. Interpreting and understanding logits, probits, and other nonlinear probability models. *Annual Review of Sociology*, 44:39–54, 2018.

[20] Max A Halvorson, Connor J McCabe, Dale S Kim, Xiaolin Cao, and Kevin M King. Making sense of some odd ratios: A tutorial and improvements to present practices in reporting and visualizing quantities of interest for binary and count outcome models. *Psychology of Addictive Behaviors*, 2021.

[21] Cristina Menni, Ana M Valdes, Maxim B Freidin, Carole H Sudre, Long H Nguyen, David A Drew, Sajaysurya Ganesh, Thomas Varsavsky, M Jorge Cardoso, Julia S El-Sayed Moustafa, et al. Real-time tracking of self-reported symptoms to predict potential covid-19. *Nature medicine*, 26(7):1037–1040, 2020.

[22] Carole H Sudre, Karla A Lee, Mary Ni Lochlainn, Thomas Varsavsky, Benjamin Murray, Mark S Graham, Cristina Menni, Marc Modat, Ruth CE Bowyer, Long H Nguyen, et al. Symptom clusters in covid-19: A potential clinical prediction tool from the covid symptom study app. *Science advances*, 7(12):eabd4177, 2021.

[23] OECD Pisa. Pisa: Results in focus. *Organisation for Economic Co-operation and Development: OECD*, 2015.

[24] R Philip Chalmers et al. mirt: A multidimensional item response theory package for the r environment. *Journal of statistical Software*, 48(6):1–29, 2012.

[25] Benjamin A Stenhaug and Benjamin W Domingue. Predictive fit metrics for item response models. *Applied Psychological Measurement*, 46(2):136–155, 2022.

[26] Maria Elena Oliveri and Matthias von Davier. Investigation of model fit and score scale comparability in international assessments. *Psychological Test and Assessment Modeling*, 53(3):315, 2011.

[27] AJ Alvero, Sonia Giebel, Ben Gebre-Medhin, Anthony Lising Antonio, Mitchell L Stevens, and Benjamin W Domingue. Essay content and style are strongly related to household income and sat scores: Evidence from 60,000 undergraduate applications. *Science advances*, 7(42):eabi9031, 2021.

[28] Rebecca Zwick and Jennifer Greif Green. New perspectives on the correlation of sat scores, high school grades, and socioeconomic factors. *Journal of Educational Measurement*, 44(1):23–45, 2007.

[29] Victor Martins Maimone and Taha Yasseri. Football is becoming more predictable; network analysis of 88 thousand matches in 11 major leagues. *Royal Society Open Science*, 8(12):210617, 2021.

[30] Dheeru Dua and Casey Graff. UCI machine learning repository, 2017.

[31] Samuel George Waugh. *Extending and benchmarking Cascade-Correlation: extensions to the Cascade-Correlation architecture and benchmarking of feed-forward supervised artificial neural networks.* PhD thesis, University of Tasmania, 1995.

[32] MM Faniqul Islam, Rahatara Ferdousi, Sadikur Rahman, and Humayra Yasmin Bushra. Likelihood prediction of diabetes at early stage using data mining techniques. In *Computer Vision and Machine Intelligence in Medical Image Analysis*, pages 113–125. Springer, 2020.

[33] Kristin P Bennett and Olvi L Mangasarian. Robust linear programming discrimination of two linearly inseparable sets. *Optimization methods and software*, 1(1):23–34, 1992.

[34] Peter D Turney. Cost-sensitive classification: Empirical evaluation of a hybrid genetic decision tree induction algorithm. *Journal of artificial intelligence research*, 2:369–409, 1994.

[35] James McDermott and Richard S Forsyth. Diagnosing a disorder in a classification benchmark. *Pattern Recognition Letters*, 73:41–43, 2016.

[36] Rajen B Bhatt, Gaurav Sharma, Abhinav Dhall, and Santanu Chaudhury. Efficient skin region segmentation using low complexity fuzzy decision tree model. In *2009 Annual IEEE India Conference*, pages 1–4. IEEE, 2009.

[37] Robert Detrano, Andras Janosi, Walter Steinbrunn, Matthias Pfisterer, Johann-Jakob Schmid, Sarbjit Sandhu, Kern H Guppy, Stella Lee, and Victor Froelicher. International application of a new probability algorithm for the diagnosis of coronary artery disease. *The American journal of cardiology*, 64(5):304–310, 1989.

[38] Beata Strack, Jonathan P DeShazo, Chris Gennings, Juan L Olmo, Sebastian Ventura, Krzysztof J Cios, and John N Clore. Impact of hba1c measurement on hospital readmission rates: analysis of 70,000 clinical database patient records. *BioMed research international*, 2014, 2014.

[39] Brendon Hall. Facies classification using machine learning. *The Leading Edge*, 35(10):906–909, 2016.
